# Social connections predict brain structure in a multidimensional free-ranging primate society

**DOI:** 10.1126/sciadv.abl5794

**Published:** 2022-04-13

**Authors:** Camille Testard, Lauren J. N. Brent, Jesper Andersson, Kenneth L. Chiou, Josue E. Negron-Del Valle, Alex R. DeCasien, Arianna Acevedo-Ithier, Michala K. Stock, Susan C. Antón, Olga Gonzalez, Christopher S. Walker, Sean Foxley, Nicole R. Compo, Samuel Bauman, Angelina V. Ruiz-Lambides, Melween I. Martinez, J. H. Pate Skene, Julie E. Horvath, Cayo Biobank Research Unit, James P. Higham, Karla L. Miller, Noah Snyder-Mackler, Michael J. Montague, Michael L. Platt, Jérôme Sallet

**Affiliations:** 1 Department of Neuroscience, University of Pennsylvania, Philadelphia, PA, USA.; 2 Centre for Research in Animal Behaviour, University of Exeter, Exeter, UK.; 3 Wellcome Integrative Neuroimaging Centre, fMRIB, Oxford, UK.; 4 Center for Evolution and Medicine, Arizona State University, Tempe, AZ, USA.; 5 School of Life Sciences, Arizona State University, Tempe, AZ, USA.; 6 Department of Anthropology, New York University, New York, NY, USA.; 7 New York Consortium in Evolutionary Primatology, NYCEP, New York, NY, USA.; 8 Section on Developmental Neurogenomics, National Institute of Mental Health, Washington, DC, USA.; 9 Department of Sociology and Anthropology, Metropolitan State University of Denver, Denver, CO, USA.; 10 Texas Biomedical Research Institute, San Antonio, TX, USA.; 11 Department of Molecular Biomedical Sciences, College of Veterinary Medicine, North Carolina State University, Raleigh, NC, USA.; 12 Department of Radiology, University of Chicago, Chicago, IL, USA.; 13 Caribbean Primate Research Center, University of Puerto Rico, Sabana Seca, Puerto Rico.; 14 Comparative Medicine, University of South Florida, Tampa, FL, USA.; 15 Department of Neurobiology, Duke University, Durham, NC, USA.; 16 Institute of Cognitive Science, University of Colorado, Boulder, CO, USA.; 17 Department of Biological and Biomedical Sciences, North Carolina Central University, Durham, NC 27707, USA.; 18 Department of Biological Sciences, North Carolina State University, Raleigh, NC, USA.; 19 North Carolina Museum of Natural Sciences, Raleigh, NC 27601, USA.; 20 Department of Evolutionary Anthropology, Duke University, Durham, NC 27708, USA.; 21 ASU-Banner Neurodegenerative Disease Research Center, Arizona State University, Tempe, AZ, USA.; 22 Department of Psychology, University of Pennsylvania, Philadelphia, PA, USA.; 23 Marketing Department, University of Pennsylvania, Philadelphia, PA, USA,; 24 Department of Experimental Psychology, Wellcome Integrative Neuroimaging Centre, Oxford, UK.; 25 Stem Cell and Brain Research Institute, Inserm, Université Lyon 1, Bron U1208, France.

## Abstract

Reproduction and survival in most primate species reflects management of both competitive and cooperative relationships. Here, we investigated the links between neuroanatomy and sociality in free-ranging rhesus macaques. In adults, the number of social partners predicted the volume of the mid–superior temporal sulcus and ventral-dysgranular insula, implicated in social decision-making and empathy, respectively. We found no link between brain structure and other key social variables such as social status or indirect connectedness in adults, nor between maternal social networks or status and dependent infant brain structure. Our findings demonstrate that the size of specific brain structures varies with the number of direct affiliative social connections and suggest that this relationship may arise during development. These results reinforce proposed links between social network size, biological success, and the expansion of specific brain circuits.

## Teaser

Brain structure is predicted by social network size but not other key social attributes in free-ranging monkeys.

## INTRODUCTION

Primates, including humans, are remarkable for their complex societies and sophisticated social cognition. Most primates form multifaceted, differentiated social relationships shaped by status, friendships, alliances, and kinship (*1*). Navigating these societies depends not only on recognizing others and responding to them appropriately but also on having information about the relationships between others and using it to make decisions (*2*). The computational demands of living in large complex social groups have been hypothesized to be a key factor driving the evolution of primate brain size (*3*). However, whether and how the diverse components of primates’ natural social lives relate to brain structure remain largely unexplored.

Macaques—particularly rhesus—are the most-studied nonhuman primate with regard to the structure and physiology of the central nervous system (*4*), with clear homologies in the human brain (*5*). In the laboratory, macaque brain structure and function correlate with group size (*6*, *7*) and social status (*8*, *9*). Although laboratory settings offer greater experimental control, they typically do not recapitulate the multidimensional societies that spontaneously emerge in free-ranging macaques. Whether and how neuroanatomical correlates of social conditions in laboratory macaques extend to naturally occurring variation in social relationships remain unknown. Here, we investigated how social relationships, both direct and indirect, affiliative and agonistic, vary with brain structure in a naturalistic population of free-ranging, group-living rhesus macaques, with minimal interference from humans.

Maintaining relationships requires recognizing individual members of the group, tracking them through space and time, and deciding when and how to interact with them. Indirect connections may arise from an understanding of third-party relationships or the global properties of the social network in which individuals are embedded (*10*). Therefore, direct and indirect connectedness may require socio-cognitive abilities reflected in the anatomy of the “social brain network” (*11*, *12*). Before structural brain imaging, we recorded affiliative and agonistic interactions among all adults (*n* = 68, females, ages 4 to 25 years) in a social group of rhesus macaques on Cayo Santiago island, Puerto Rico (see Materials and Methods; fig. S1). From macaques’ most overt affiliative interaction—grooming—we derived the group’s social network and computed both direct (i) and indirect (ii to iv) measures of social connectedness for each animal over 4 years old, including (i) number of partners, or “social network size”; (ii) ability to act as a bridge between disconnected parts of the social network, or “betweenness”; (iii) distance to every other member of the group, or “closeness”; and (iv) connectedness of their partners, or “eigenvector centrality” (*13*). From paired agonistic interactions (e.g., threats, chases, or submissions), we derived each adult’s social status (see Materials and Methods)—a key facet of social capital that shapes competitive acquisition of resources. Subsequently, we collected magnetic resonance imaging (MRI) anatomical scans of the left hemispheres of all individuals in the social group {*n* = 103, ages 1 month to 25 years [6.89 5.46 (mean SD)]; table S1}, including juveniles and infants for which we did not collect direct behavioral observations (*n* = 35). From the 103 individuals scanned, our main analysis focused on the 68 adults for which we collected behavioral data before scanning (fig. S1).

## RESULTS

### Social network size predicts mid–superior temporal sulcus and ventral dysgranular insula volume

First, we quantified regional differences in brain structure between individuals using deformation-based morphometry (DBM) analysis of the anatomical scans of the adults’ left hemisphere (see Materials and Methods). DBM measures relative individual variation in local volume and has been implemented across a range of species and protocols, including investigations of social cognition (*14*) and pathology (*15*). To study the relationship between sociality and adult brain structure, we considered two generalized linear models. First, we focused on social variables widely used as predictors of success in both human (*16*) and nonhuman animals (*17*), namely, social network size (or unweighted degree) and social status. Second, we tested the association between brain structure and higher-order components of an individual’s position in the social network. We focused on three indirect measures of connectedness that describe each individual’s polyadic position in the social network: closeness, betweenness, and eigenvector centrality. In both models, age, sex, and whole-brain weight were included as covariates. Social status was also included as a covariate in the second model (table S4). We ran a mass univariate analysis, testing each voxel in the gray matter independently (*n* = 230,773). In all models, the outcome variable was the log-transformed determinant of the Jacobian matrix for each voxel, a value representing the amount a given voxel needed to be expanded or compressed to match the group-average brain (see Materials and Methods). Note that a brain from our sample was used as a study template to build the group-average brain and was subsequently excluded from the analyses; thus, sample size was *n* = 67.

**Fig. 1.**
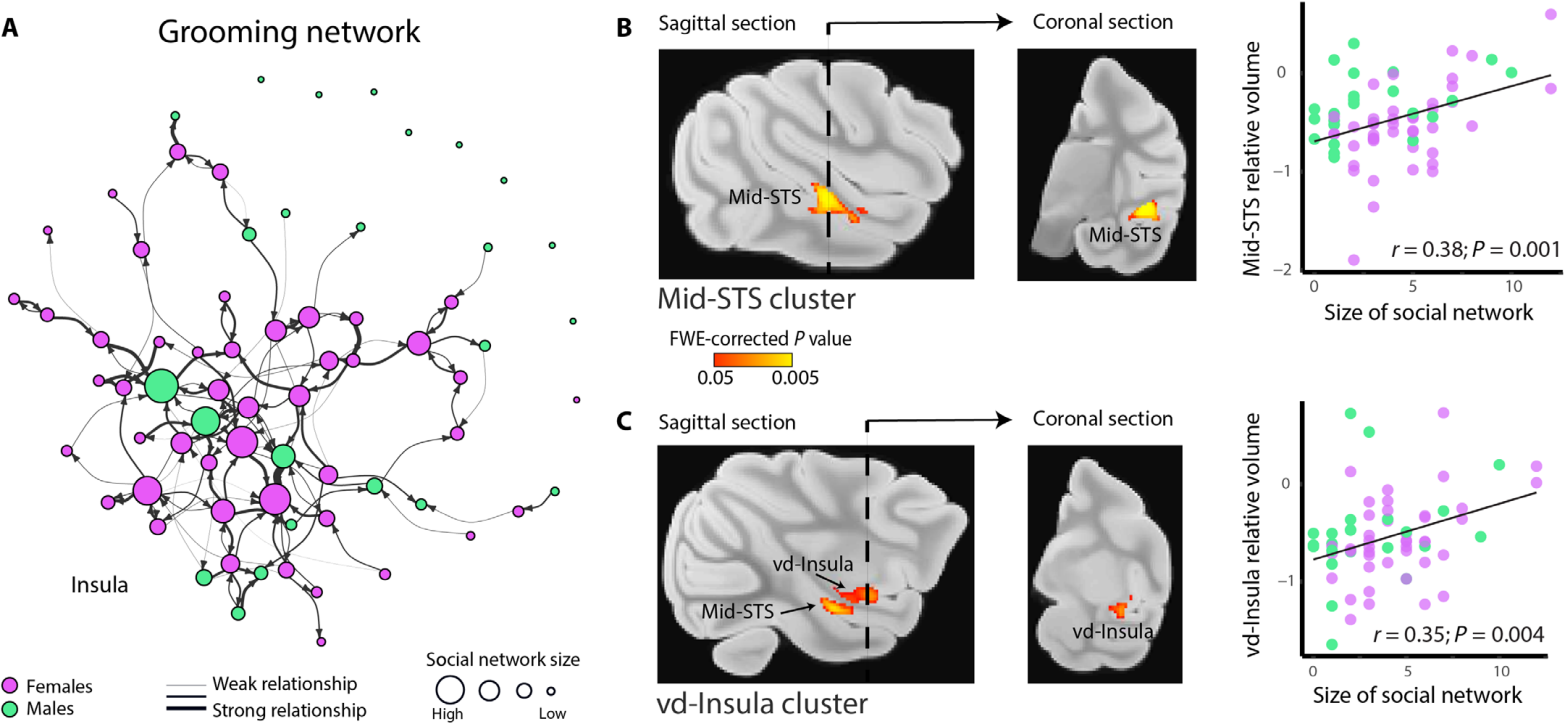
Social network size correlates with mid-STS and ventral dysgranular insula relative volume in free-ranging rhesus macaques. (**A**) Weighted grooming network for Cayo Santiago social group HH, from which the left cerebral hemispheres were collected and scanned using structural MRI (T_1_-weighted images). Females, purple; males, green. Arrows indicate a directed grooming relationship, and arrow thickness represents interaction strength (time observed grooming corrected for total time observed). Measures of indirect connectedness were derived from this weighted sociogram, and we used unweighted degree as a measure of direct connectedness. (**B**) Mid-STS cluster sagittal and coronal views (*P* < 0.05, FWE-corrected) and plot of the relationship between social network size and average log-transformed Jacobian value of the mid-STS cluster (a measure of relative volume; see Materials and Methods; *r* = 0.38, *P* < 0.005). (**C**) Same as (B) for ventral dysgranular insula (vd-Insula) cluster (*P* < 0.05, FWE-corrected; *r* = 0.35, *P* < 0.005). The sagittal section shown includes part of the mid-STS cluster. To avoid confusion, we labeled all clusters visible on the sagittal and coronal sections. See fig. S5 for more coronal views of the significant clusters. See fig. S10 for a comparison of results when excluding seven individuals with ambiguous social status (see Materials and Methods for more details). Note that correlation plots in (B) and (C) were run to provide illustrations of the effects detected using voxel-wise multivariate modeling (see Materials and Methods).

Social network size positively correlated with the relative volume of the mid–superior temporal sulcus (STS) and ventral dysgranular insula (vd-Insula) [Fig. 1, fig. S5, and table S3; *P* < 0.05, family-wise error (FWE)–corrected for whole-brain volume; see Materials and Methods]. The mid-STS cluster (size, 149.2 mm^3^), located in STS associated areas IP (IPa), PG (PGa), and the rostral Temporo-parieto-occipital area (TPOr) (*18*), matches previous studies relating experimentally induced variation in group size to brain structure in laboratory macaques (*6*). In monkeys, functional MRI studies have shown that this region responds selectively during observation of social scenes (*19*), and electrophysiological studies have demonstrated that single neurons in the mid-STS monitor other actions (*20*) and encode abstract strategic information and social context in monkeys choosing to cooperate or compete with a partner (*21*). On the basis of its computational and anatomical properties, this region has been identified as the precursor of the neural architecture supporting mentalizing, an ability central to social cognition in humans (*22*, *23*). The second cluster is primarily located in an antero-ventral part of the dysgranular subdivision of the insula (*24*) areas Id and Pi from the Saleem and Logothetis atlas (*25*), or areas dm, dv, and ap based on the architectonic organization of the insula proposed by Evrard and colleagues (*26*). This region supports the expression of affiliative behavior in rhesus macaques (*27*) and empathy in humans (*28*, *29*). Despite being associated with distinct social processes (*12*), the mid-STS and the vd-Insula are monosynaptically connected (*30*), suggesting functional interaction between these two regions. Thus, the size of each monkey’s social network—the number of friendships and alliances they maintain—selectively predicts the size of specific interconnected brain structures previously shown to mediate higher-order social information processing. Last, a small cluster (9.2 mm^3^) was also observed in the vicinity of the dorsal subdivision of the lateral amygdala extending into the ventral lateral part of the putamen (fig. S5 and table S3), areas previously implicated in processing the emotional significance (*31*) and value (*32*) of social information.

The relationship between social network size and gray matter morphology can be further investigated by decomposing total network size into in-degree centrality (i.e., the number of partners who groom an individual) and out-degree centrality (i.e., the number of partners an individual grooms). In a follow-up analysis, we replaced social network size with unweighted in-degree and out-degree in the “social network size and social status” model. Using a mass univariate whole-brain approach (i.e., gray matter mask, *n* = 230,773), we did not find a significant effect of in-degree or out-degree with threshold-free enhancement clustering (TFCE) correction for multiple comparisons. With a more liberal threshold (uncorrected *P* < 0.001 over >100 contiguous voxels), an effect of in-degree was detected within the mid-STS, fully overlapping with the social network size mid-STS cluster. No significant voxels for in-degree were detected outside the mid-STS, and no effects were detected with out-degree (fig. S7A). These findings indicate that the relationship observed between grooming network size and mid-STS morphology might be partially mediated by the number of partners who groom an individual monkey, but not by the number of individuals that a monkey grooms.

### Social status and indirect connections do not predict brain structure

**Fig. 2.**
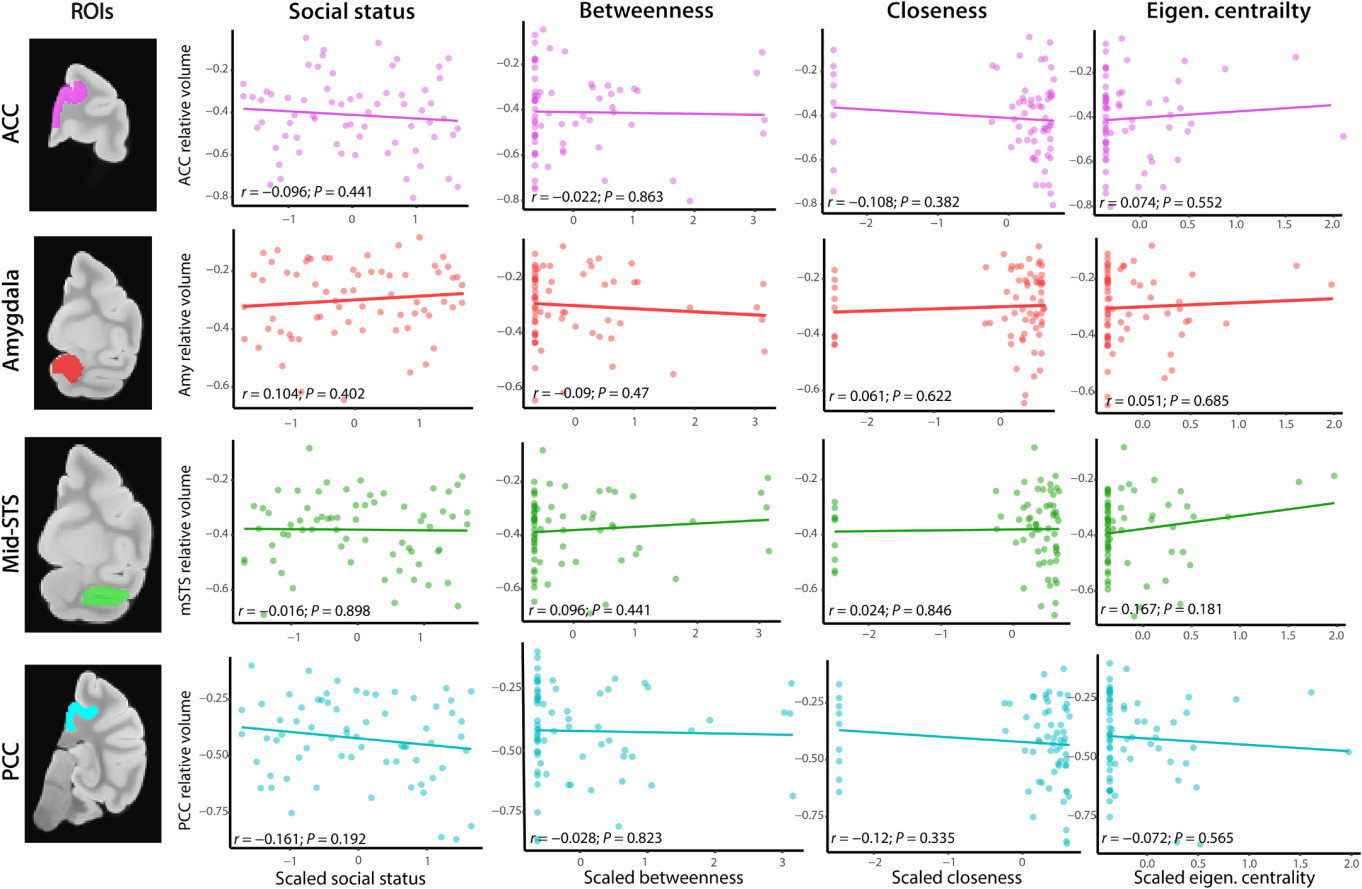
Social status and indirect measures of connectedness do not correlate with the size of four key areas in the social brain network. Correlation plots between average Jacobian value of four social brain network ROIs [rows, bottom to top: PCC, light blue; mid-STS, green; amygdala (Amy), red; ACC, pink] and four social parameters (columns left to right: social status, betweenness, closeness, and eigenvector centrality; scaled measures) in 67 adult macaques. None of the correlations are significant. A few individuals in our sample were completely disconnected from the group’s social network (Fig. 1A), resulting in undefined indirect connectedness measures (*65*). We ran our models setting the indirect connectivity of those individuals to 0, causing the bimodal distribution of closeness. Excluding disconnected individuals (*n* = 9) did not change our results qualitatively (table S5). We tested three additional ROIs based on previous findings in laboratory animals relating brain structure to social status (*6*, *8*): hippocampus, brainstem, and striatum (see Materials and Methods). We did not find a significant relationship with social status in our sample in these additional ROIs after correcting for multiple comparisons. Eigen. centrality, eigenvector centrality; *r*, correlation coefficient; *P*, uncorrected *P* value. Similarly to Fig. 1, note that correlation plots are provided to illustrate the observed absence of effects.

Unexpectedly, we found no significant associations between brain structure and other social characteristics previously shown to predict fitness in primates, including social status (*9*) and indirect connectedness (*13*). These findings persisted even when restricting analysis to four key areas of the social brain network defined a priori: amygdala, STS, posterior cingulate cortex (PCC), and anterior cingulate cortex (ACC) (Fig. 2, correlation plots for illustrative purposes). By contrast, laboratory studies have found that brain structure varies with social status in macaques (*6*, *8*). Differences in how social status is computed and the social environment between free-ranging macaques and those in the laboratory may explain this difference. For a more direct comparison with previous laboratory studies, we calculated a “submission-dominance index” (SDI), a laboratory proxy for social status, which is based on the percentage of dominant interactions out of all social interactions per individual [see Materials and Methods; (*8*)]. Again, we did not detect a relationship between brain morphology and SDI. In free-ranging conditions, the percentage of dominance interactions (i.e., won agonistic interactions) out of all interactions rarely surpassed 20%, with a peak around 7%, whereas in laboratory conditions the distribution of scores was wider and SDI can reach 100% (fig. S9). This difference reflects a higher occurrence of affiliative interactions in free-ranging conditions relative to laboratory ones. Macaques in captivity are typically managed in pairs or small groups within a confined space and do not self-assort into groups. This limits their ability to avoid or escape dominant individuals, and restricts opportunities for assistance from an extended social network (*33*). For these reasons, social status may affect the health, physiology, and neuroanatomy of captive macaques more strongly than it does in free-ranging macaques. These considerations highlight the importance of investigating biological variation in naturalistic settings, which can differ substantially from laboratory environments (*33*).

### Infant brain structure is not predicted by the mother’s social network size

**Fig. 3.**
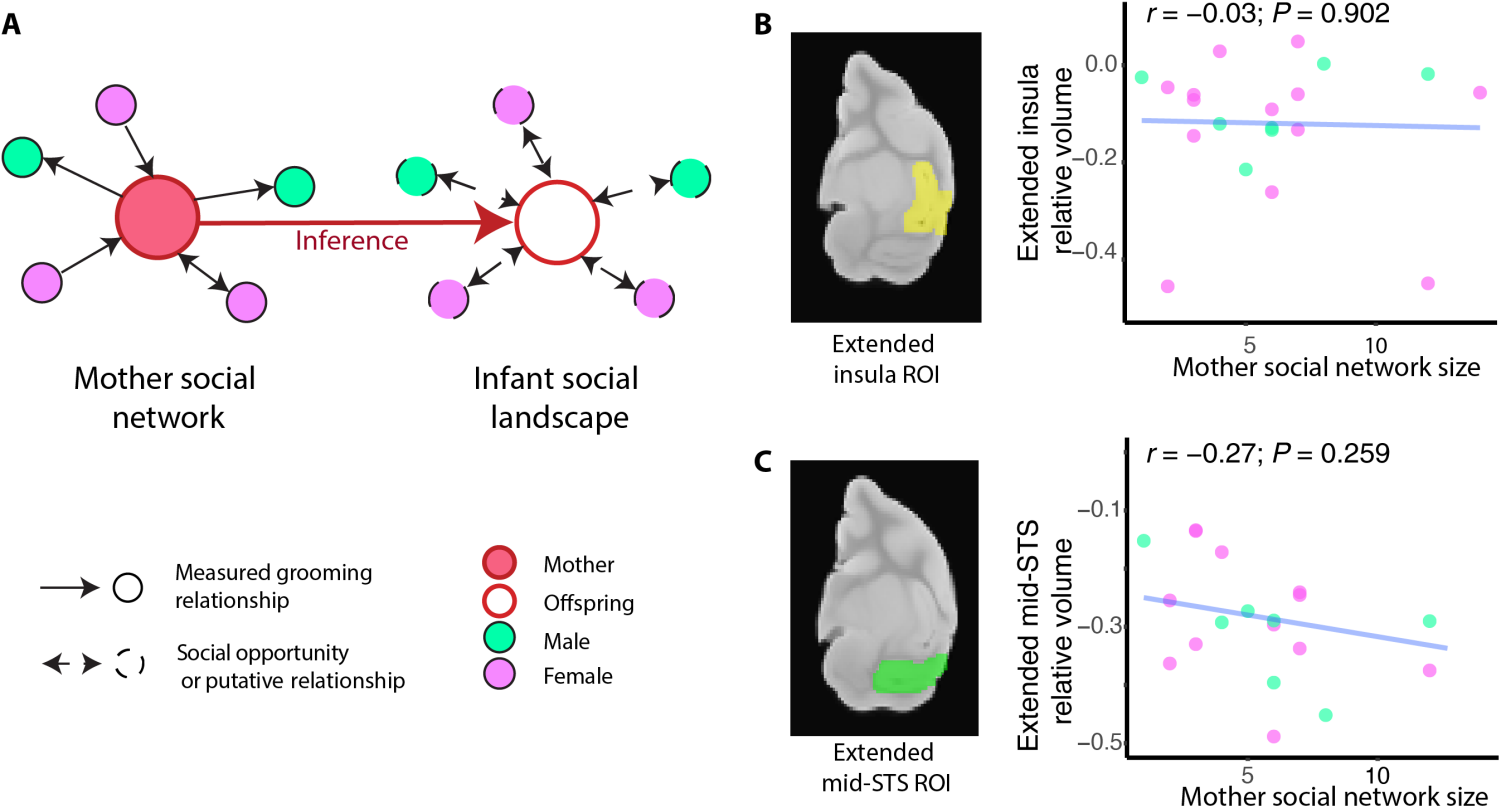
Mother’s social network size does not correlate with mid-STS or insula relative volume in dependent infants. (**A**) Mother’s social network was used to infer their infant’s social landscape and was computed from grooming interactions. (**B**) The relative volume of dependent infants’ insula and surrounding cortex (i.e., average log-transformed Jacobian values in extended insula ROI) did not correlate with mother’s social network size, (**C**) nor did the relative volume of infants’ mid-STS and surrounding cortex. Similarly to Figs. 1 and 2, correlation plots are illustrations of the observed absence of effects. Because our sample of infants was smaller than that of adults, we conducted a second analysis in which the “Insula-midSTS” mask was restricted to only the significant voxels in the adult analysis (*n* = 4605 voxels). We did not find an effect of the mother’s social network or status in the infants using this smaller ROI mask. Last, we detected a significant correlation between social network size and the determinant of the Jacobian in the mid-STS of infants’ mothers (*n* = 20, FWE-corrected *P* value for the extended Insula-midSTS mask, *n* = 17,158 voxels).

Our sample also included dependent infants and juveniles, allowing us to investigate whether brain structure covaries with the social environment during development. Although we did not collect behavioral data on these young individuals, dependent infants (up to 5 months old) spend most of their time in proximity to their mother [100% at 1 month and 75% at 5 months; (*34*)] and have partner preferences similar to their mother’s preferences (*35*), and their interactions are shaped by their mother’s social status (*36*) (more details provided in Materials and Methods). Therefore, we used the mother’s social network size and status to infer the infant’s social milieu—specifically the number of individuals an infant was likely to encounter and the quality of those encounters. Juveniles, on the other hand, particularly males, spend time apart from their mother and are not necessarily exposed to the same social network, unlike infants. Because we could not infer the social networks and social status of juveniles from the social networks and status of their mothers with satisfying accuracy, we excluded juveniles (*n* = 14) from subsequent analyses. To test for the relationship between infant brain structure (*n* = 21, 13 females) and maternal social status and network size, a separate DBM was done, using an infant brain as reference to accommodate for the large difference in brain size between adult and dependent infants (fig. S3; see Materials and Methods). Note that this reference brain was excluded from the analyses such that the sample size for the dependent infant analysis was *n* = 20. Our models included infant age, sex, and brain weight as covariates. On the basis of our findings in adults, we restricted our analysis to two ROIs (regions of interest) centered on the mid-STS and vd-Insula (*n* = 17,158 voxels). We found no relationships between the relative size of these brain areas and maternal social network size or status (Fig. 3).

Two caveats in this analysis should be considered. First, we did not directly measure infants’ social networks, and instead relied on the mother’s social network to infer the infants’ social milieu, or the number of distinct individuals that infants likely encountered and the quality of those interactions. While it is clear that mothers exert substantial influence over their infant’s social landscape (*34*–*37*), especially up to 20 weeks of age (the age of the oldest infant in our sample), some uncertainty remains regarding the relationship between mother’s social network size and the infant’s social milieu. Nonetheless, previous studies indicate that infant rhesus macaques spend 75 to 100% of their time in close proximity to their mothers over the first 5 months of life (*35*), and maternal relationships predict 64% of daughters’ affiliative relations at ~5 months (*37*). It is thus reasonable to surmise that the number of monkeys an infant encountered or interacted with was shaped by the size of their mother’s social network, and yet we found no relationship between maternal social network size and infant brain structure. Second, our sample size for this analysis was only about one-third of that of the adult analysis (*n*_infant = 20 versus *n*_adult = 67), which may be insufficient power to detect an effect in infants of similar magnitude as found in adults. This power issue was at least partly mitigated by our ROI-based approach, which included only 17,158 voxels (~7% of the voxels used in the adult whole-brain analysis). To address this issue in a complementary way, we ran the social network size and social status model only including the infants’ mothers (*n* = 20) with the same “Insula-midSTS” mask used in infants. We again observed an effect of social network size on midSTS in mothers after correcting for multiple comparisons using TFCE.

## DISCUSSION

Here, we show that social network size predicts the size of specific areas in the social brain network in free-ranging rhesus macaques, building upon and extending previous studies in laboratory macaques and humans (*6*, *7*). Our findings confirm social network size as a key social variable related to mSTS volume, irrespective of whether monkeys live outdoors in large open-field conditions or indoors in a smaller laboratory colony. Moreover, the effect of an individual’s social network size on the brain could not be reduced to its subcomponents, in-degree and out-degree centrality. In free-ranging conditions, social network size captures the total number of differentiated relationships an individual maintains—a multi-dimensional social construct that includes directionality (received versus given) and familiarity (close friends versus acquaintances) of relationships, as well as kinship (kin versus non-kin). Our results suggest that this multidimensional construct better predicts individual variation in brain structure than any component does on its own. When comparing the effect of relationship directionality, we found that in-degree centrality was a relatively bigger driver of variation in the size of the mid-STS than was out-degree—in accordance to previous work in humans (*38*). This differential sensitivity to incoming versus outgoing interactions might reflect the primacy of an egocentric frame of reference for representing social interactions and managing an individual’s relationships (*39*). The preponderance of mid-STS neurons encoding self’s strategy over other’s strategy during a cooperation game supports this hypothesis (*21*).

Unexpectedly, we found no neuroanatomical correlates of social status or higher-order features of social network topology such as the connectedness of indirect relationships [i.e., “friends of friends” (*13*)]. Our results suggest the cognitive processes required to maintain and navigate direct relationships exert the strongest computational demands on the brain and therefore predict variation in brain structure. In natural settings, status is inherited in rhesus macaque females (*40*) and obtained largely by queueing instead of direct contest in males (*41*). Thus, acquiring and maintaining social status may not require sophisticated social cognition reflected in gross variation in gray matter morphology. Similarly, an advantageous polyadic position in the social network, such as a high eigenvector centrality (*13*), can arise and be maintained via simple behavioral rules that do not require advanced social cognition (*42*). Moreover, direct relationships may be more relevant for biological success in this population, for example, by mitigating the negative consequences of adversity (*43*). Notably, markers of immunity also vary more strongly with direct social connections than with social status or indirect connections in this same population (*44*). Other aspects of neuroanatomy, such as white matter tract integrity, have revealed a complementary picture of the relationship between sociality and brain structure in humans (*45*, *46*), and future studies in macaques should address these possibilities.

Environmental factors might also influence the extent to which dimensions of sociality relate to brain structure. For instance, proxy measurements for social status in the laboratory correlate with amygdala volume and neuronal activity in rhesus macaques (*8*, *31*). Constraints on available space and the absence of self-assortment into groups in laboratory settings could explain differences in the relative importance and physiological impact of social status and social network size. For example, the higher incidence of affiliative behavior observed in free-ranging compared to laboratory settings (fig. S9) could explain the effect of number of grooming partners on the vd-Insula—involved in the expression of affiliative behavior in macaques (*27*) and empathy in humans (*28*, *29*)—which was not observed in previous laboratory-based studies (*6*).

Dependent infants in our sample (1 to 5 months old) spend most of their time [75 to 100%; (*34*)] in proximity to their mother, and their social milieu is strongly shaped by their mother’s social network and status (*34*, *35*, *37*). However, unexpectedly, we did not detect an effect of the maternal social environment on infant mSTS and vd-Insula morphology. This finding suggests that network size–related structural differences in adults are not detectable early in life and may arise at a later developmental stage. These results are consistent with previous work on social brain development in rhesus macaques. Specifically, face-selective areas in the macaque temporal cortex emerge over the first year of development (*47*), in parallel with developmental changes in visual attention to social stimuli (*48*), as infants begin to groom (*49*), and interact with partners other than their mother (*35*). Future studies using direct observations of infants’ interactions and a larger sample size across development will permit a more powerful test of the relationship between social development and brain structure.

Our findings inform accounts of primate brain evolution that emphasize complex social environments as a selective force driving neocortex expansion. Group size, classically used as a proxy for social complexity, is a poor descriptor of the social environment individuals experience within groups (*50*). Using more granular measures, our results endorse the hypothesis that the number of relationships one must maintain to maximize fitness (*51*) propels the expansion of specific brain circuits (*52*, *53*), both across and within species.

## MATERIALS AND METHODS

### Subjects

We studied the behavior and collected brain tissues from a group of rhesus macaques living in a semi–free-ranging colony on Cayo Santiago Island, Puerto Rico (18°09 N, 65°44 W). The colony has been continuously monitored since it was established in 1938 following the release of 409 animals originally captured in India (*54*). Cayo Santiago is managed by the Caribbean Primate Research Center (CPRC), which supplies food to the population daily and water ad libitum. There is no contraceptive use and no medical intervention aside from tetanus inoculation when animals are weaned yearlings. Animals are free to aggregate into social units, and disperse to new ones, as they would in the wild. There are no natural predators on the island.

The average annual growth rate of the Cayo Santiago rhesus macaque population is far faster than that of wild populations, making population control necessary through capture and removal of animals (*55*). Starting in 2016, the CPRC selectively removed entire social groups as a population management strategy. To maximize the research potential from the CPRC’s activities, the Cayo Biobank Research Unit (CBRU) was created to collect and archive postmortem tissues from animals removed, including brains. The data in this study come from this CBRU database, specifically brain tissues from 2016. All procedures related to capture, removal, and euthanasia were conducted by the CPRC in accordance with protocols approved by the animal use committee of the University of Puerto Rico (protocol number 338300).

Subjects for this study were a single social group composed of 103 individual monkeys, 39 males and 64 females, ages 1 month to 25 years (see fig. S1 and table S1 for age and sex distribution). Each monkey was individually recognizable by tattoos, ear notches, and facial features. We used observational data from mid-July to mid-October 2016 to characterize the social phenotypes and map social networks of individual monkeys age 4 years and older before trapping (*n* = 68).

### Behavioral data collection

Behavioral data were collected using 5-min focal animal samples (*56*) on Teklogic Psion WorkAbout Pro handheld computers, with Noldus Observer software. Focal sampling was done following a previously established protocol (*57*). Briefly, we recorded the occurrence, duration, and partner identity of all affiliative (e.g., grooming and passive contact) and agonistic (e.g., aggression, threats, submissions, and displacements) social interactions with all social partners age 4+. At the beginning and end of each focal follow (0- and 5-min mark), we collected instantaneous scan samples to record the state behavior of the subject (grooming, feeding, resting, and traveling), the number of juveniles (ages 1 to 3) within 2 m (i.e., in proximity), and the identity of all adults (age 4+) in proximity. We balanced collection of focal samples on individuals across time of day (a.m. versus p.m.) to account for temporal variation in behaviors.

We collected grooming, proximity, and aggression data on all individuals age 4 and above from group HH in the 3 months preceding their removal from the island, during the birth season (mid-July to mid-October 2016). We recorded on average (SD) 1.46 (0.08) hours of focal follows, or 17.6 (0.96) focal samples, per individual on 68 of our 103 subjects. The remaining subjects were less than 4 years old.

### Computing social status and social network metrics

#### *Social status*

On the basis of literature in rhesus macaques supporting sex differences in how social status is acquired, dominance rank was determined separately for males and females (*58*). Females are philopatric and form maternally inherited stable linear dominance hierarchies, where daughters acquire rank just below their mothers (*36*, *59*). In contrast, males typically disperse from the natal group and acquire a rank in the new group largely based on their duration of tenure (*60*, *61*). To determine social status in both sex, we used the direction and outcome of win-loss agonistic interactions recorded during focal animal samples or during ad libitum observations (e.g., threats, displacements, contact and noncontact aggression such as bite and chase, and submissive interactions). Known maternal relatedness was used to resolve behavioral gaps in the female hierarchy (*58*, *62*, *63*). To account for group size, social status was defined by the percentage of same-sex individuals that a subject outranked, and ranged between 0 and 100 (0 = lowest social status, i.e., they outranked 0% of same-sex individuals; 100 = highest social status, i.e., they outranked 100% of same-sex others). Note that our data were largely consistent with a transitive linear hierarchy: In females, only 8 of 161 (<5%) dominance interactions violated transitivity, and in males, only 2 of 85 (<3%). In total, 5 of 44 females and 2 of 24 males included in this study had ambiguous dominance, i.e., observed dominance interactions violated the transitivity assumption. Excluding these seven adults from our analyses did not change our results qualitatively (fig. S10). A conjunction analysis revealed an 82.58% overlap between the clusters observed in the two analyses.

#### *Calculating social network metrics*

In this study, group HH’s social network was based on grooming interactions between individuals age 4+. From grooming interactions, we built an interaction matrix where each column and row corresponded to an individual. Each matrix entry contained the standardized time two individuals were observed grooming each other (i.e., divided by the amount of time a given pair was observed). We then used the R package “igraph” (*64*) to plot the group’s weighted sociogram (Fig. 1A) as well as to compute the measures of direct and indirect connectedness: (i) social network size (function degree in igraph), or the number of grooming partners; (ii) in-degree, or the number of partners that initiate grooming with the individual; (iii) out-degree, or the number of partners an individual initiates grooming with; (iv) betweenness (betweenness function in igraph), a measure of whether an individual acts as a broker in its social network, or connects disconnected parts of its network; (v) closeness (function closeness in igraph), a measure of distance with all other individuals in the network; and (vi) eigenvector centrality (eigen_centrality function in igraph), a measure of the popularity of one’s connections, or in other words do one’s friends themselves have many friends? All measures of indirect connectedness were weighted, whereas social network size, in-degree, and out-degree were not.

### Brain tissue collection

Immediately following veterinary euthanasia, brains were perfused with physiological saline, removed from the cranium without the meninges (fig. S2), weighted (using an Ohaus Adventurer 600g scale, to the decigram resolution), and sectioned sagittally into left and right hemispheres. The right hemisphere was cryopreserved and not used for this study. The left hemisphere was placed in 10% formalin to preserve the tissue for imaging.

### Structural brain imaging data collection

Brain samples were placed in a container with Fluorinert FC3283 (3M) and then degassed before being placed in a human head coil. Six to eight hemispheres were scanned simultaneously.

In postmortem brains, conventional T_1_-weighted structural protocols did not produce satisfying gray/white matter contrast; therefore, we used a three-dimensional balanced SSFP (steady-state free precession) pulse sequence previously adapted for scanning formalin-preserved postmortem samples (*65*). The TRUFI (true fast imaging with steady-state free precession) sequence chosen produced high gray/white matter contrast, albeit inverted compared with conventional T_1_-weighted structural acquisitions (gray matter has high relative signal; white matter, low).

### Preprocessing of structural brain images

TRUFI data are sensitive to B_0_ inhomogeneities, resulting in banding artifacts (stripes of low signal). To account for this, data were acquired in “phase-cycled” pairs (0° or 180°) in which the regions of low signal in one dataset have high signal in the other. An average structural dataset was produced from all structural scans, effectively removing banding artifacts.

FSL BET tools (*66*) were used to create brain masks, which were manually adjusted by blind “examiners.” Then, we masked the T_1_ structural images to extract a “structural brain” image (i.e., which only includes the brain) for each individual. Those images were then used for a DBM analysis.

### DBM analysis of structural MRI images

Structural data were analyzed with a voxel-based morphometry style approach using FSL tools FNIRT and Randomize (*67*, *68*). The logic of the approach is that if a group of brain images can be warped to a near-identical image, then volumetric changes involved in that warping process give a measure of the local differences in brain structure between individuals. This method has been validated by comparing results with volumetric measurements of particular structures. For instance, Gaser and colleagues (*69*) identified a strong correlation between the voxel-wise determinant of the Jacobian, the dependent measures obtained from a DBM analysis, and the ventricular/brain ratio based on manual delineation on MRI scans. DBM also provides sensitive and reliable markers of disease progression in patients suffering from frontotemporal dementia (*15*).

Because the TRUFI sequence used inverted structural image signal values, we could not use the standard rhesus macaque Montreal Neurological Institute (MNI) template as our reference brain, as done in previous studies (*6*, *8*). Instead, we built a study-specific template. All the brains were first aligned to a reference brain selected randomly from our sample, followed by nonlinear registration using FNIRT (*67*). The resulting images were averaged to create the study-specific template. The native gray matter images were then nonlinearly reregistered to this template. This process underwent five iterations, each time increasing the resolution of the warps and refining the template. The final iteration was performed with a warp resolution (knot spacing of cubic B-splines) of 1 mm. The determinant of the Jacobian of the warp field was then extracted—the Jacobian is a matrix of the directional stretches and compressions required to register one image to another, and the determinant of this matrix gives a scalar value for the volumetric change implied (henceforth “Jacobian value”). The Jacobian values were then used as the dependent variable in the statistical analyses (see the following section). The reference brain used for registration was excluded from these analyses, resulting in a sample size of *n* = 67.

Note that the Jacobians values in some regions were not distributed around the value “1,” which is the average value for the studied population, taking into account the initial registration to the reference subject used here. Therefore, the reader should remember that Jacobian value is a relative measurement.

### Generalized linear models

#### *Social network size and social status model*

We focused on social variables widely used as predictors of biological success in both human (*16*) and nonhuman primates (*17*), namely, social integration and social status. Social integration often includes the number and/or the strength of relationships as measures. In our data, strength of connections and number of partners correlated highly (*r* > 0.9) such that we only included one of the two metrics. We chose “number of partners” (i.e., social network size) as our measure of social integration because recent work has shown that Cayo Santiago macaques widened their social network rather than reinforced their relationships in the aftermath of a natural disaster (*71*), suggesting that the size of one’s network is critical for alleviating adversity in this population. The dependent variable in this analysis (and in subsequent analyses) was the log-transformed Jacobian value for each voxel, which encodes the local volume difference between the reference and subject image (see the previous section). Jacobian values are bounded by zero and unbounded above such that it is common practice to apply the logarithmic transform (*70*). The independent variables in this model included social network size and status and the following covariates: age, sex, and whole-brain weight. Given that we scanned only the left hemisphere of our subjects (the right hemisphere was flash-frozen for later gene expression and regulation analyses), whole-brain weight measured after extraction and perfusion was used as a proxy for whole-brain volume. To confirm the validity of this proxy, we measured the correlation between whole-brain weight and left hemisphere volume and found that they were tightly correlated (*r* > 0.9, *P* < 2.2 × 10^−16^; fig. S4).

On the basis of the socioecology of rhesus macaques, we computed social status separately for males and females, and used matrilineal structure to resolve gaps in the female hierarchy. However, previous laboratory studies investigating the relationship between social status and brain morphology in rhesus macaques computed a proxy for social status (*8*) that differs in important ways with the rank measure used in this study. This proxy combines agonistic and submissive interactions across both males and females and does not account for matrilineal structure. Therefore, to permit direct comparison with previous laboratory studies, we computed an SDI—the percentage of dominant interactions with both males and females out of the total social interactions per individual—and recomputed the social network size and social status model replacing social status with SDI. This supplemental analysis included all 67 individuals included in the main analyses.

#### *“In-degree and out-degree” model*

We reran the social network size and social status models replacing grooming network size (undirected degree) with in-degree (i.e., number of unique partners that groom the individual) and out-degree (i.e., number of unique partners that the individual grooms). Variables included in this model correlated weakly to moderately (*r* < 0.5; fig. S6). Note that rank does not correlate highly with in-degree in this population (*r* = 0.39). We used a whole-brain approach (i.e., gray matter mask, *n* = 230,773); we did not find a significant effect of in-degree or out-degree after correcting for multiple comparisons using TFCE (“randomize” function in FSL, 5000 permutations). To investigate potentially weaker effects that did not survive correction for multiple comparisons across the whole brain, we lowered our statistical threshold to uncorrected *P* < 0.001 over >100 contiguous voxels (>12.5 mm^3^).

#### *“Sex and social status interaction” model*

Social status in rhesus macaques is experienced differently in females and males. Females inherit their status from their mother, while males disperse to a new group when they reach sexual maturity (age 5 to 6) and thereafter social status correlates with group tenure length (*40*). This distinction could have led to opposite anatomical relationships with social status for each sex, yielding an absence of a main effect for social status. To address this issue, we ran a follow-up model testing the interaction between sex and social status. The model included sex, social status, and the interaction between sex and social status as experimental variables, and age and brain weight as control variables (table S4); all variables are fixed effects. We found no relationship between brain structure and social status or the interaction between sex and social status at the whole-brain level, nor using an ROI approach.

#### *“Topology of social landscape” model*

This model focused on testing the relationship between an individual’s indirect connectedness to other individuals in its social network and gray matter morphology. Experimental variables in this model were closeness, eigenvector centrality, and betweenness of the individual (see the “Calculating social network metrics” section for more details). We also included age, sex, brain weight, and social status as control variables in the model (table S4).

A few individuals in our sample were completely disconnected in the group’s social network (Fig. 1A), which resulted in undefined indirect connectedness measures (*64*). It is not uncommon in this study system to have males be fully disconnected in their social network (*71*, *72*), and eight of the nine disconnected subjects are males. We ran our models setting the value of these individuals to 0 (Fig. 2) and again excluding them all together (table S5). The results remained qualitatively the same in both cases.

Note that social network size (number of partners) and indirect connectedness measures (especially closeness and betweenness) correlate moderately to strongly in our dataset (*r* > 0.5; fig. S6). Because of a concern for multicollinearity, we did not run a full model combining all aforementioned terms. All the variables included in the same models correlated weakly to moderately (*r* < 0.4; fig. S6 and table S4).

#### *Whole-brain analysis*

We started with a whole-brain analysis approach, focusing on gray matter throughout the brain. We used a gray matter mask created using the FSL tool FAST on the study-specific template. Using the “randomize” function from FSL (5000 permutations), we ran the three models described above for each voxel in this mask (*n* = 230,773) and corrected for multiple comparisons using the TFCE algorithm (*73*). The resulting family-wise–corrected p-map was thresholded at *P* = 0.05. Figure 1 (B and C) shows statistically significant clusters. Location and size of significant clusters are found in table S3.

#### *ROI analysis*

To ensure that negative results obtained when testing voxels throughout the brain were not due to low power, we took an ROI approach. We focused on the gray matter of key structures of the social brain network defined a priori: the amygdala, the ACC, the PCC, and the hippocampus. We ran the three models described above for each voxel in this “social brain” mask (*n* = 23,261 voxels) and corrected for multiple comparisons using TFCE.

To further test the hypothesis that insufficient statistical power limited our ability to detect an effect of social status specifically, we tested three additional ROIs based on previous findings in laboratory animals relating brain structure to social status (*6*, *8*): hippocampus (*n* = 4843 voxels), brainstem (including Raphe nuclei and hypothalamus; *n* = 5793 voxels), and striatum (*n* = 13,140 voxels). We also retested the amygdala on its own (*n* = 2462 voxels). We did not find a significant relationship with social status in our sample in these additional ROIs after correcting for multiple comparisons using TFCE.

#### *Analyses on dependent infant brains*

To investigate whether brain structure varies with the social environment before monkeys are socially independent of their mothers, we ran a DBM analysis on the brains of dependent infants (ages 1 to 5 months old). This analysis (registration and calculation of Jacobian values) was done separately in infants and adults because of the significant difference in brain size between the two groups (fig. S3). The DBM analysis was performed in the same way as in adults (see the “DBM analysis of structural MRI images” section), using an infant brain from our sample to build the study-specific template. This template infant brain was excluded from the analysis such that the sample size in the dependent infant analyses was *n* = 20.

We did not have direct behavioral data for these infants and instead used their mother’s social network and rank to index variation in the infants’ social environments. Dependent infants spend most of their time in close proximity to their mothers [100% at 1 month, ~75% at 5 months; (*34*)], clinging onto their mother’s belly to travel and breastfeed. Infants rely entirely on their mother to provide nutrition in the first 4 months of their life and are fully weaned between the ages of 12 to 14 months (*74*). The oldest infant in our sample was 4.8 months old (fig. S1). Given the amount of time infants spend in close proximity to their mothers, they have many opportunities to observe and interact with their mother’s partners. Mothers also serve as models for social interactions: By observing their mothers being amicable toward close relatives and avoidant or aggressive toward nonrelatives, infants learn how to behave toward relatives and nonrelatives accordingly and develop kin-biased social networks (*75*). Thus, important components of the formation of social relationships in young infant macaques involve being passively exposed to the mother’s environment and the mother’s behavior (*76*). As a result, the social milieu to which infants are exposed is strongly shaped by their mothers’ social network from 1 week until at least 30 weeks of age (or 7 months old) (*35*). More specifically, infants have similar partner preferences in terms of relatedness (i.e., kin bias), dominance, age, and sex as their mother (*35*, *75*), even at 30 weeks of age when they are more often away from their mother, and maternal relationships predict 64% of daughters’ affiliative relations at ~5 months (*37*). Last, in rhesus macaques, offspring acquire their mothers’ dominance rank early in life through the agonistic support they receive from mothers and through observations of their mother’s behavior toward other individuals (*77*–*79*). On the basis of the extensive literature on maternal influence on social development in rhesus macaque [see (*36*) for review], including in the very same macaque population under study here (i.e., Cayo Santiago macaques), infants were assigned their mother’s social network size (or number of grooming partners) and social status as an index of their social landscape.

The model for dependent infants included mother’s social status and social network size as experimental variables, and infants’ age, sex, and brain weight as control variables (table S4). We performed an ROI approach focusing on brain areas that contained significant clusters in adults, namely, the insula and the mid-STS. We ran a model for each voxel in this Insula-midSTS mask (*n* = 17,158 voxels; ~7% of whole-brain gray matter mask was used in adults) and corrected for multiple comparisons using TFCE. Note that the sample size in the dependent infant analysis is less than a third of the sample size in the adult analysis (*n*_infant = 20 versus *n*_adult = 67), which reduces our power to detect an effect. However, the ROI-based approach we used should at least partly mitigate lower sample size. To further address this issue, we restricted the Insula-midSTS mask to only the significant voxels in the adult analysis (*n* = 4605 voxels). Results remained qualitatively unchanged. We also artificially reduced the power of the adult analysis by running the social network size and social status model only in the mothers of infants (*n* = 20), using the same mask as for the infant analysis (Insula-midSTS mask, *n* = 17,158 voxels). Once again, we detected an effect of social network size, but not social status, among mothers. Last, we tested whether age effects on infant brain morphology might be occluding social effects. We did not find significant age effects after controlling for multiple comparisons within our ROIs (mSTS and Insula), even when we restricted our search to the significant voxels from the adult analysis. This gives us confidence that the absence of an effect of mother’s social network size or status on infant mSTS and insula is not due to age.

### Controlling for kinship structure

We collected brain samples from a single social group of macaques where animals were related to each other. This may constitute an important statistical issue whereby individual subjects are not independent from each other, leading to a potentially inflated false-positive rate (*80*). To ensure that kinship was not inflating our estimates of social network size, we modeled log-transformed Jacobian values using efficient mixed-model association (EMMA) models to control for relatedness among individuals in our dataset (*81*). EMMA models were implemented in the EMMREML package in R and included the same variables as in the “social network size and status” model. The EMMA algorithm fits a linear mixed model with population structure or relatedness included as a random effect. To generate the required relatedness matrix, we ran synbreed (*82*) using the extensive population-wide Cayo Santiago pedigree and generated an additive numerator relationship matrix. We ran EMMA models separately for each voxel in our gray matter mask (*n* = 230,773) and used a false discovery rate–corrected threshold of *P* = 0.05 (*83*) for statistical significance. This method of correcting for multiple comparisons is more stringent than the widely used random-field theory (RFT)–based approach in neuroimaging. It does not account for image smoothness as RFT does (*84*) such that we expected a more restricted cluster than in our initial analysis using the “Randomize” tool in FSL. The resulting significant cluster for social network size overlaps extensively with the cluster found using the FSL-based analysis (81.98% of the pixels in the EMMA cluster overlap with the FSL-based cluster; fig. S8), which suggests that relatedness is unlikely to be biasing or driving our effects.

## Supplementary Materials

### This PDF file includes:

Cayo Biobanking Research Unit members Figs. S1 to S11 Tables S1 to S5 References

## Notes

View/request a protocol for this paper from *Bio-protocol*.
