# Supplementary material for: Social connections predict brain structure in a multidimensional free-ranging primate society
Source: Sci Adv. 2022 Apr 13;8(15):eabl5794. doi: 10.1126/sciadv.abl5794 (PMC9007502; doi:10.1126/sciadv.abl5794)
Supplement: Supplementary file 1 — Cayo Biobanking Research Unit members Figs. S1 to S11 Tables S1 to S5 References [file sciadv.abl5794_sm.pdf]

Science Advances
AAAS

Supplementary Materials for

Social connections predict brain structure in a multidimensional free-ranging primate society

Camille Testard*, Lauren J. N. Brent, Jesper Andersson, Kenneth L. Chiou, Josue E. Negron-Del Valle, Alex R. DeCasien, Arianna Acevedo-Ithier, Michala K. Stock, Susan C. Antón, Olga Gonzalez, Christopher S. Walker, Sean Foxley, Nicole R. Compo, Samuel Bauman, Angelina V. Ruiz-Lambides, Melween I. Martinez, J. H. Pate Skene, Julie E. Horvath, Cayo Biobank Research Unit, James P. Higham, Karla L. Miller, Noah Snyder-Mackler, Michael J. Montague, Michael L. Platt, Jérôme Sallet*

*Corresponding author. Email: camille.testard@pennmedicine.upenn.edu (C.T.); jerome.sallet@inserm.fr (J.S.)

Published 13 April 2022, *Sci. Adv.* **8**, eabl5794 (2022)
DOI: 10.1126/sciadv.abl5794

This PDF file includes:

Cayo Biobanking Research Unit members
Figs. S1 to S11
Tables S1 to S5
References

Members of Cayo Biobank Research Unit:
Lauren J.N. Brent[1], James P. Higham[2], Melween I. Martinez[3], Michael J. Montague[4], Michael L. Platt[4,5,6], Noah Snyder-Mackler[7,8,9]
[1] Centre for Research in Animal Behaviour, University of Exeter, Exeter, UK
[2] Department of Anthropology, New York University, NY, USA
[3] Caribbean Primate Research Center, University of Puerto Rico, PR
[4] Department of Neuroscience, University of Pennsylvania, Philadelphia, PA, USA
[5] Departments of Psychology, University of Pennsylvania, Philadelphia, PA, USA
[6] Departments of Marketing, University of Pennsylvania, Philadelphia, PA, USA
[7] Center for Evolution and Medicine, Arizona State University, AZ, USA
[8] School of Life Sciences, Arizona State University, AZ, USA
[9]ASU-Banner Neurodegenerative Disease Research Center, Arizona State University; Tempe, AZ, USA

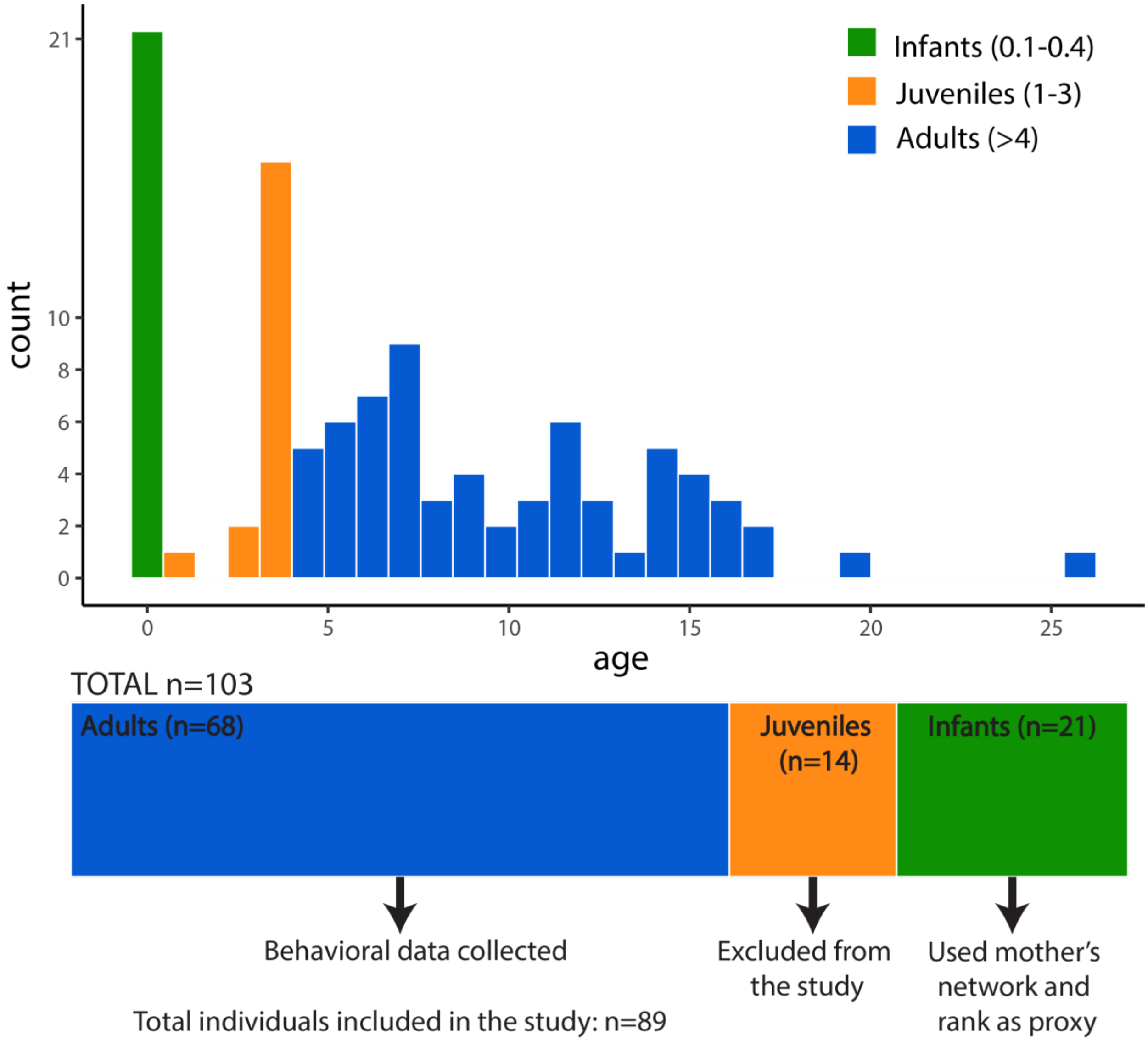

Figure S1. **Study subject description**. **Top panel**: Age distribution of our sample color-coded by age category. Green: infants, Orange: Juveniles; Blue: Adults. **Bottom panel**: Schematic description of subject population. Juveniles were excluded from the study because we did not collect direct behavioral data on them and could not infer their sociality from their mother, unlike infants.

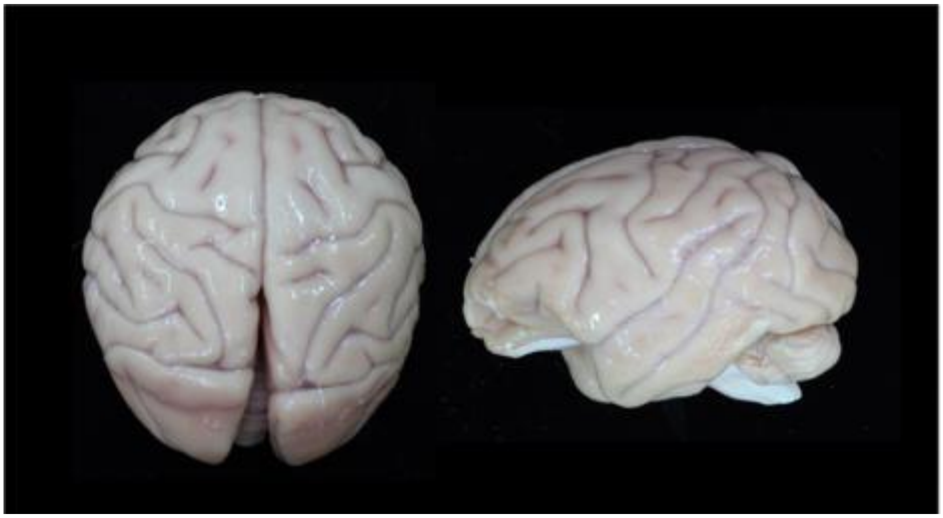

Figure S2. Photo of rhesus macaque brain after saline perfusion and cranium extraction.

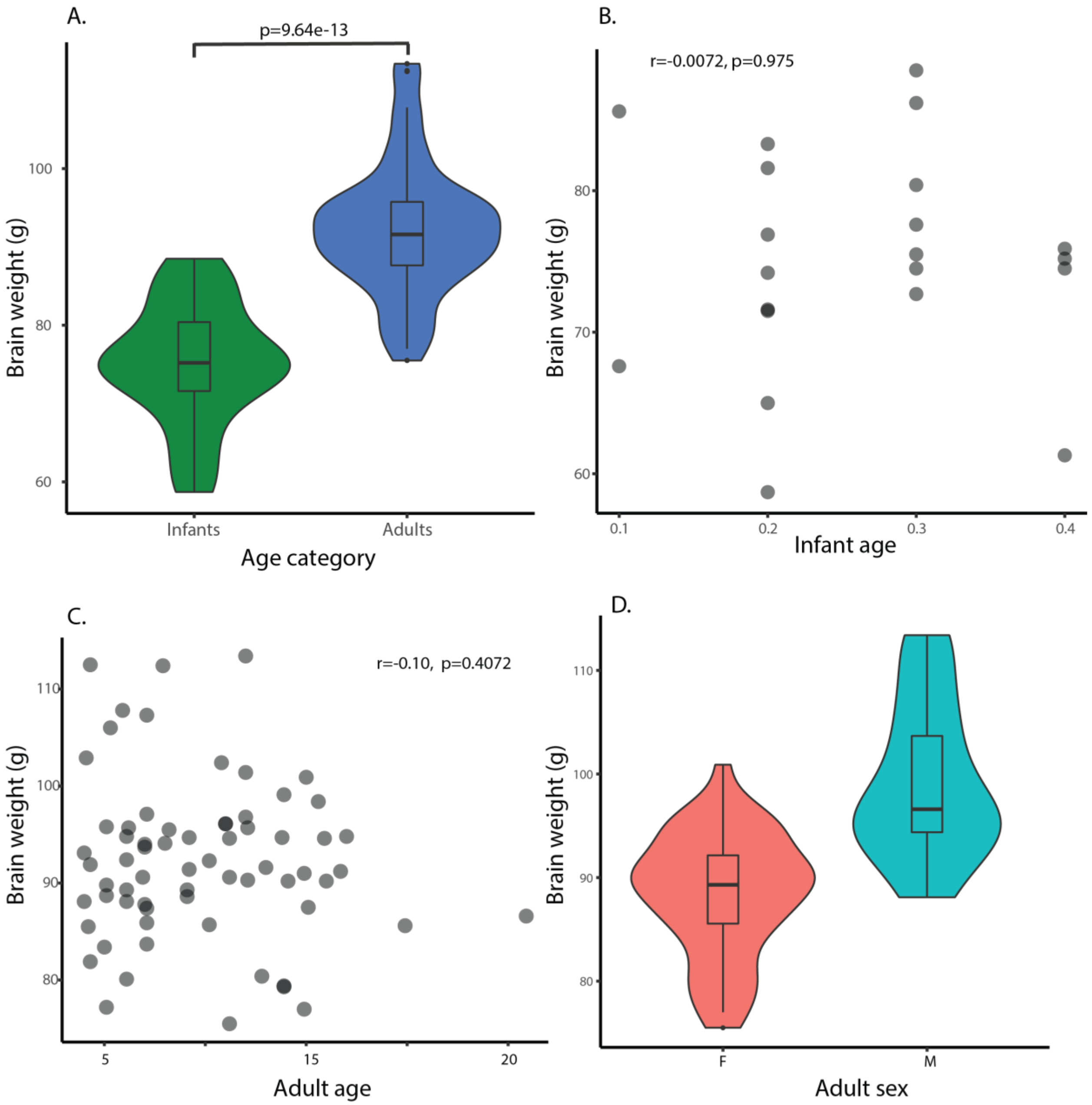

Figure S3. **Relationship between age and brain weight**. (A) Brain weight (in grams) differs between infants and adults. (B) Scatter plot of infant age and brain weight (age in years), color-coded by sex (red: females, blue: males). We found no significant correlation between the two variables (uncorrected p-value, spearman correlation). (C) Scatter plot of adult age and brain weight color-coded by sex. Again, we found no significant correlation between the two variables (uncorrected p-value, spearman correlation). (D) Brain weight separated by sex in adults. On average males have heavier brains than females.

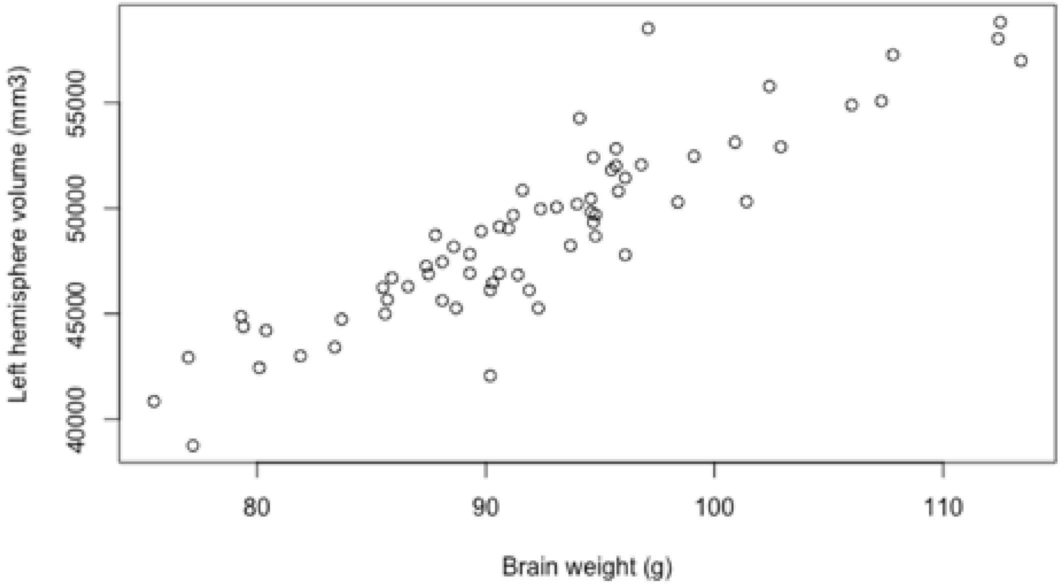

Figure S4. Left hemisphere volume correlates tightly with whole brain weight (r=0.90, p<2.2e-16).

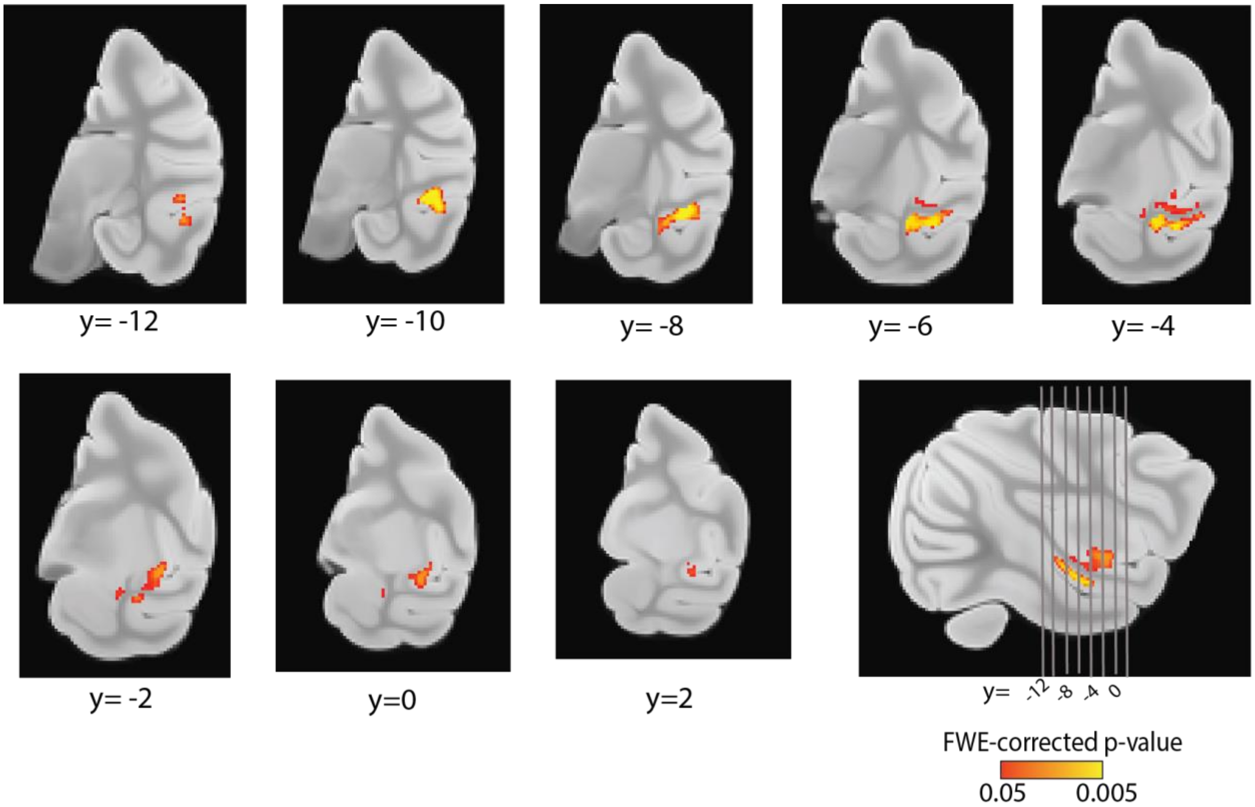

Figure S5. Coronal views of the "social network size" cluster from the "Social network size and social status" model. Coordinates relative to the AC in the template anatomical space. The location of AC in the template anatomical space is x=39, y=60, z=47. STS = Superior Temporal Sulcus; vd-Insula = ventral dysgranular Insula; Lat. = Lateral; Put = Putamen; Amy = Amygdala.

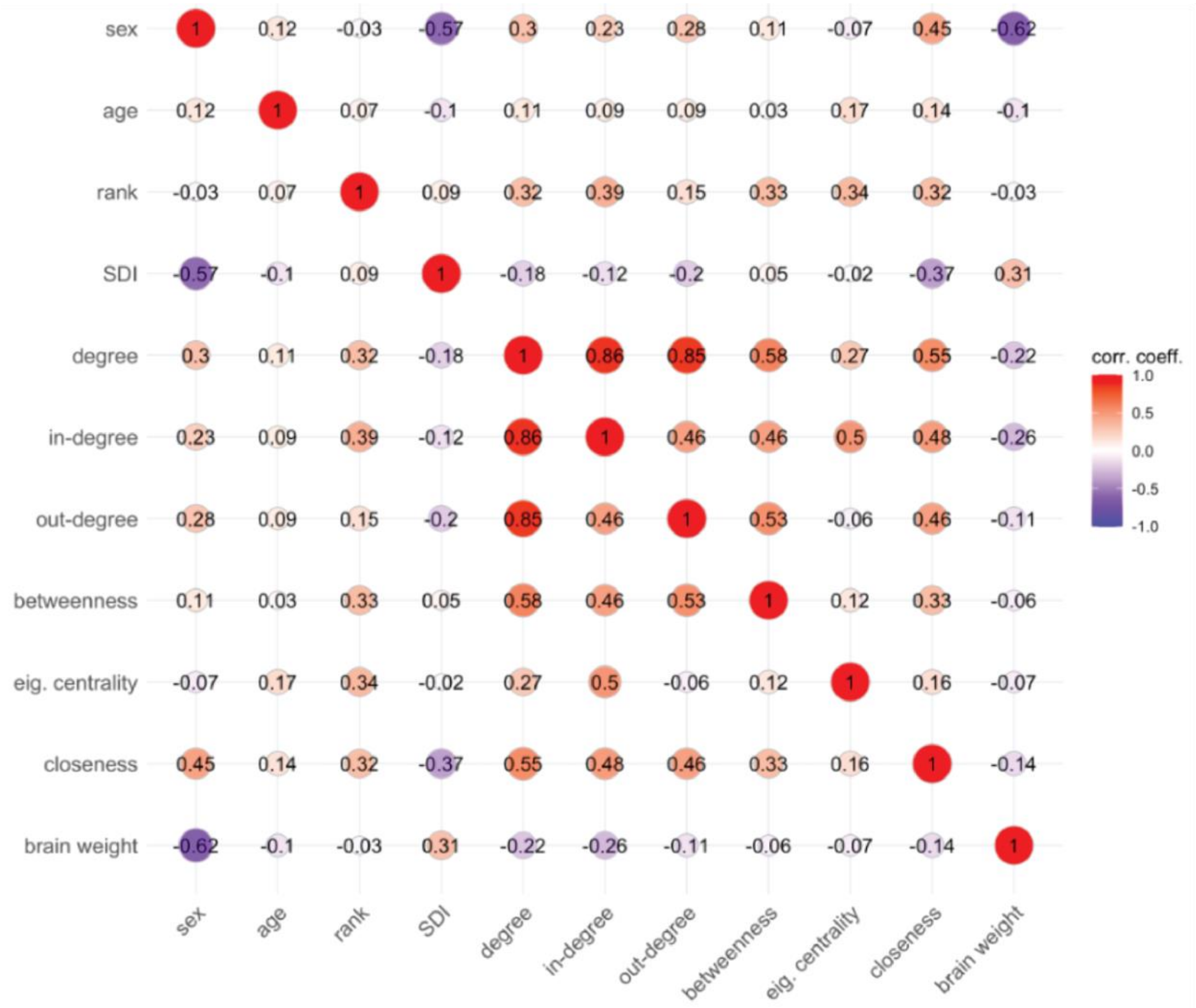

Figure S6. Correlation plot of all continuous variables included in this study. See Table S4 for details about which variables were evaluated together. Plotted using 'ggcorrplot' function in R. Degree, in-degree and out-degree are all unweighted measures. Betweenness, eigenvector centrality and closeness are weighted. Rank = social status; Degree = social network size; eig. centrality = eigenvector centrality; SDI = Social-Dominance Index (laboratory proxy for rank).

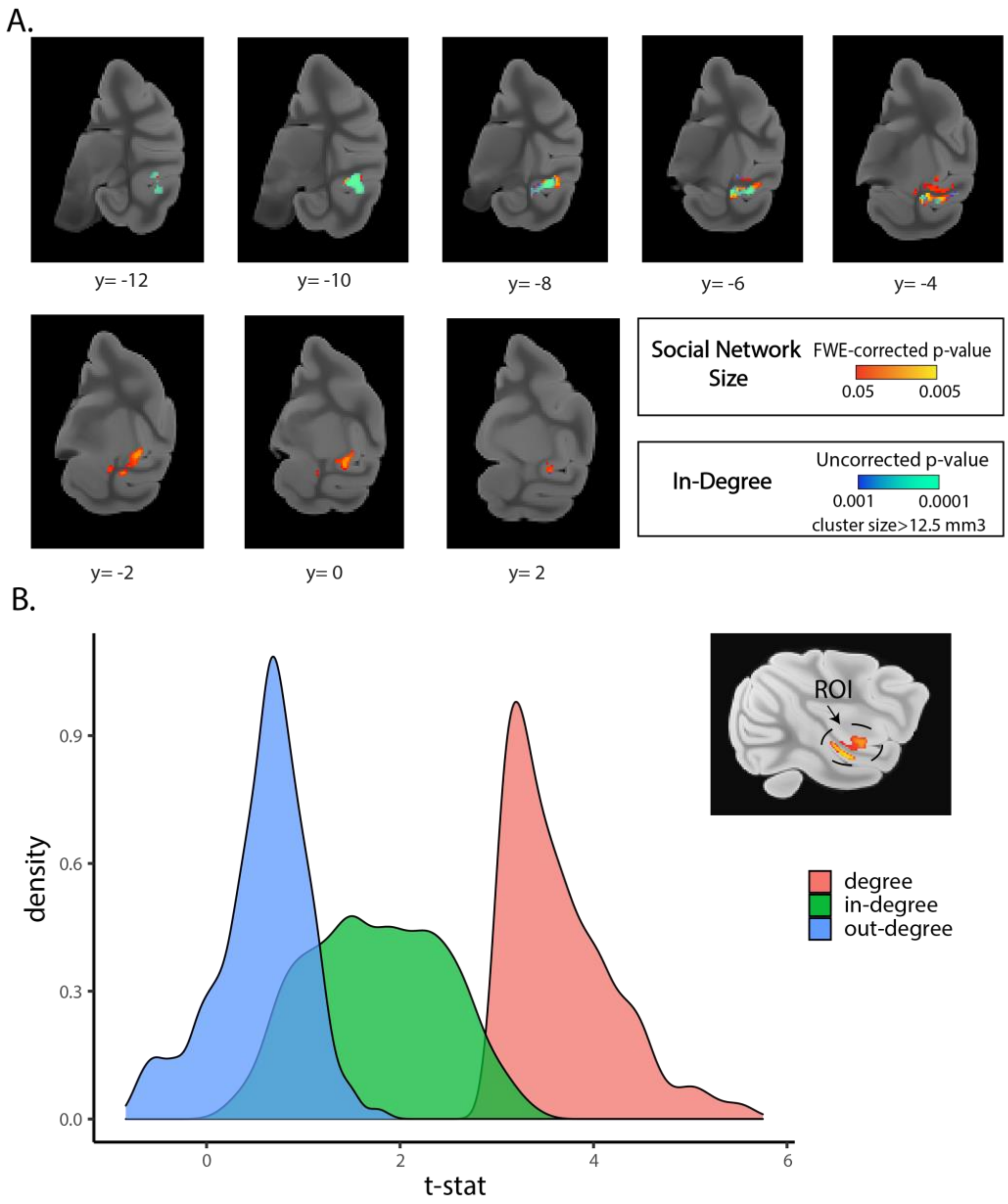

Figure S7. (A) Coronal views of the in-degree cluster from the "In-degree and out-degree" model, overlapping with the "social network size" cluster from the "social network size and social status" model. Coordinates relative to the AC in the template anatomical space. (B) Density plot of voxel-wise t-statistics (or standardized estimates) within the "social network size" cluster ROI. STS = Superior Temporal Sulcus; vd-Insula = ventral dysgranular Insula; Lat. = Lateral; Put = Putamen; Amy = Amygdala; ROI = Region of Interest.

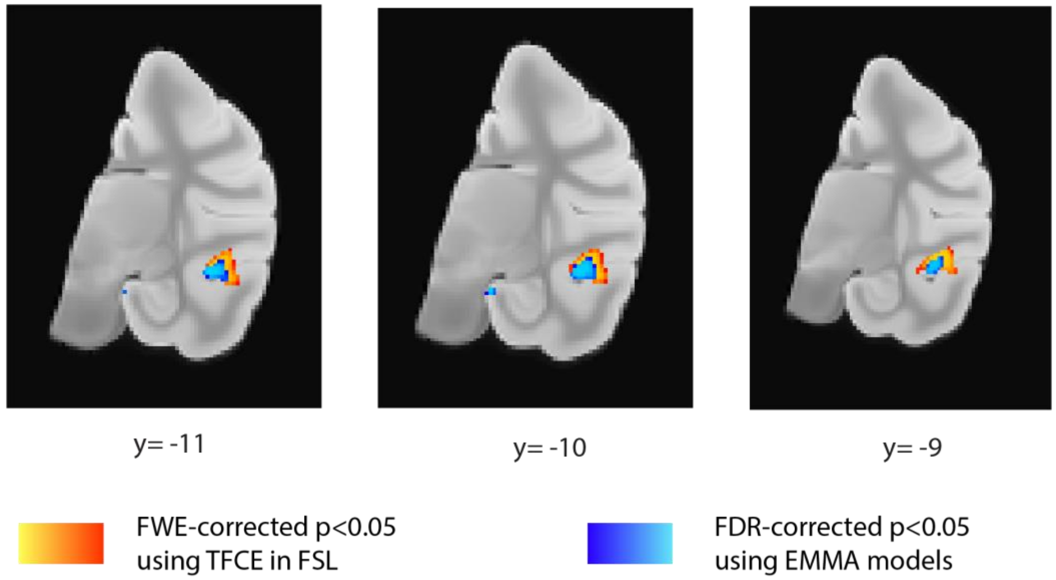

Figure S8. **Overlap between TFCE-corrected GLM thresholded p-maps (FWE-corrected p<0.05) and FDR-corrected EMMA models p-maps (corrected p<0.05)**. Three slices are represented and demonstrate the overlap between the clusters with the two approaches. Y coordinates are given relative to the anterior commissure.

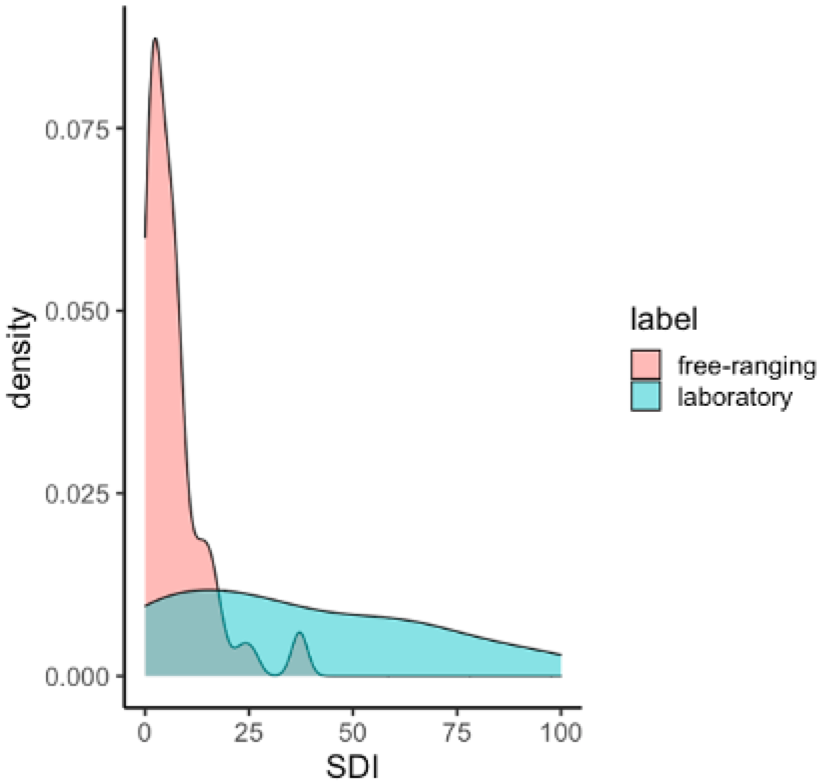

Figure S9. Density plot of the “Submission-Dominance Index” in laboratory macaques from Noonan and colleagues(*8*) and free-ranging macaques from this study.

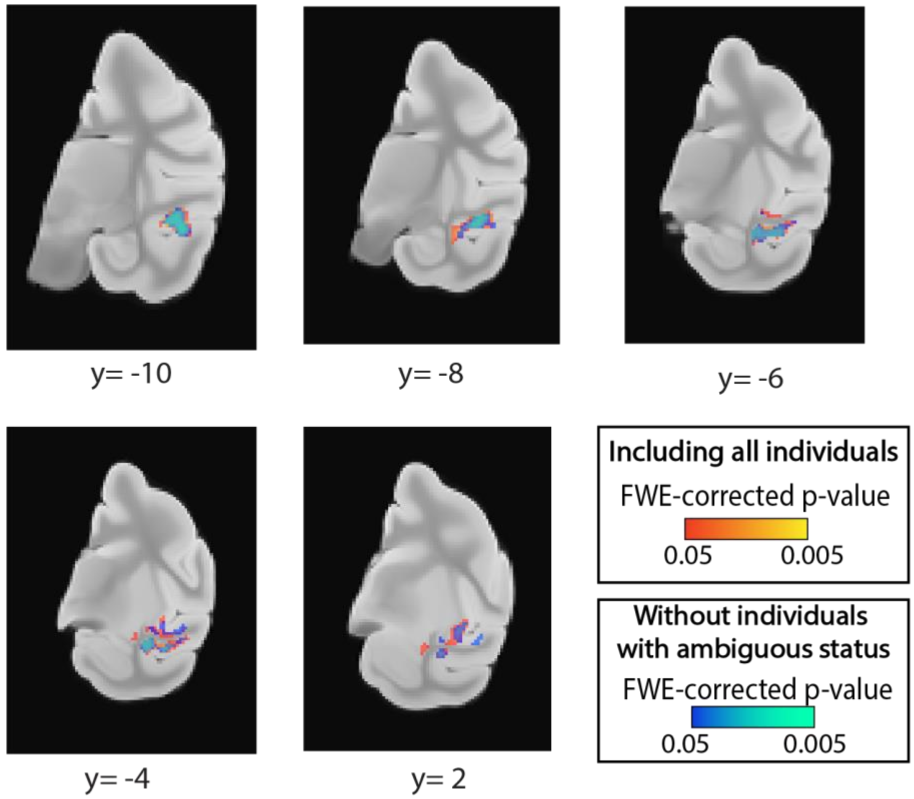

Figure S10. Statistically significant effects of social network size in the mSTS and vd-Insula whether individuals with ambiguous social status (n=7) were excluded or not (clusters overlap by 82.58%). P-value corrected for multiple comparisons using TFCE, as in Figure 1.

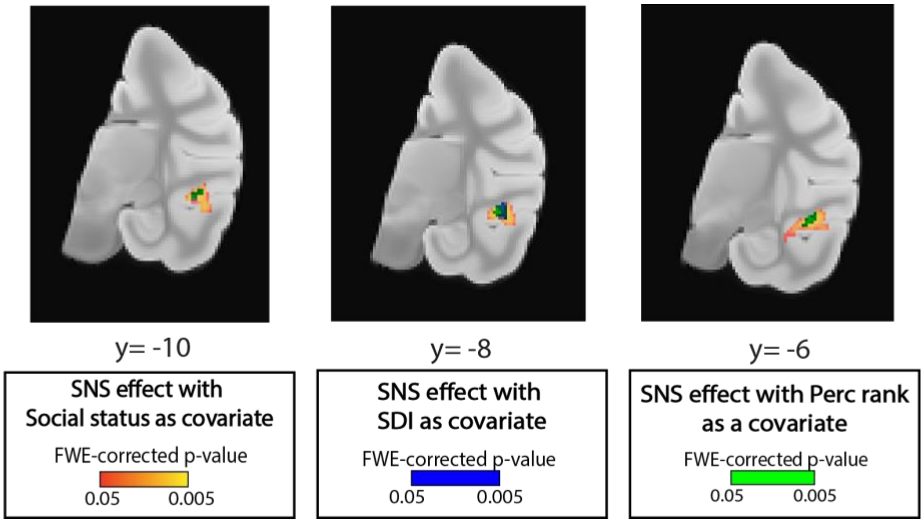

Figure S11. Absence of dominance effect on gray matter morphology but significant effect of social network size in mSTS, irrespective of dominance measure used (social status, SDI or Perc). "Social status" refers to the social status measure used in all main analyses in this paper, which is calculated separately for males and females and is based on direct agonistic interactions, transitivity and matriline rank in females. "SDI" (Social Dominance Index), refers to the index used in previous laboratory animal studies which have linked hierarchy to brain structure(*6*, *8*). SDI is the percentage of dominant interactions (i.e., wins in agonistic interactions) with both males and females out of *all* social interactions (both affiliative and agonistic), for each individual. It does not assume transitivity or consider matriline information. "Perc" is a rank estimate computed using the Perc package in R, which calculates dominance point estimates and uncertainty only using agonistic interactions and the transitivity assumption (*no* matriline information), combining males and females. This network-based method, called Percolation and Conductance (*85*), uses information from both direct and indirect dominance pathways to calculate consistency in the direction of transitive dominance pathways from monkey A to monkey B (e.g. via pathway through C, D, and/or E). "Social status" and "Perc" metrics correlated highly ($r=0.807$, $p<0.0001$), while SDI was uncorrelated with the other two ($r<0.1$). Irrespective of the dominance metric used, we fail to find an effect of social status after correcting for multiple comparisons in a whole-brain analysis (n=67 individuals; 230,773 voxels). Nevertheless, we still find significant effects of social network size in the mSTS regardless of the dominance metric used ($p<0.05$ FWE-corrected for whole-brain).

SEX \ AGE	Dependent Infants (<6mo)	Juveniles [1 - 4)	Young Adults [4-9)	Mature Adults [9-15)	Old Adults [15-19)	Geriatric (19+)	TOTAL
**Males**	8	7	14	6	4	0	39
**Females**	13	7	18	22	2	2	64
**Total**	21*	14	32**	28**	6**	2**	103

Table S1. Age-sex distribution of our subjects based on the life history of Rhesus macaques *(86)*. For age intervals, brackets "[" mean the number is included in the interval, and parentheses ")" mean the number is *not* included. *Young infants considered for DBM analysis presented on Fig 3; **Adults subjects included.

**Scanner**	3T Trio
**Coil**	8 Channel Human Head coil
**Resolution**	0.5mm isotropic
**Steps /Phase-cycle**	0, 180deg
**Averages**	>10 per phase-cycle

Table S2. Summary of structural brain image data collection parameters.

Cluster name	CoG coordinates (x,y,z) in template anatomical space	CoG coordinates (x, y, z) *relative to AC* in template anatomical space	Cytoarchitectonic area based on Saleem and Logothetis Atlas	Size in mm3
Antero-Ventral insula	22.8, 58.1, 39.0	-16.2, -1.9, -8	Dysgranular insula, area Id extending ventrally into the parainsular cortex (Pi)	68.8
mid-STS	21.4, 52.8, 37.2	-17.6, -7.2, -9.8	Cluster within IPa, PGa and TPOr	149.2
Lateral Putamen/Lateral Amygdala	28.4, 57.5, 36.6	-10.6, -2.5, -10.4	Cluster with the caudal part of the dorsal subdivision of the lateral Amygdala extending caudally within the most ventral and lateral part of the putamen	9.6

Table S3. Location (x,y,z relative to the Anterior Commissure) and size (mm3) of significant clusters. We chose the Anterior Commissure (AC) as our reference for the coordinates. The location of AC in the template anatomical space is x=39, y=60, z=47. Coordinates are given with decimal resolution. We mapped the center of gravity onto the Saleem and Logothetis atlas(*25*). CoG = Centre of Gravity.

Subjects	Adults (≥4 yrs old)				Dependent infants (<5 months old)
**Models**	**Social network size and status**	**In-degree & out-degree**	**Sex and social status interaction effect**	**Indirect connectedness**	**Mother social network size and status**
**Experimental variables**	Social status (or SDI, or Perc-rank)	Out-degree	Sex	Betweenness	Mother's social status
	Social network size	In-degree	Social status	Eigenvector centrality	Mother's social network size
			Social status:Sex	Closeness	
**Control variables**	Age, sex, brain weight	Age, sex, brain weight, social status	Age, brain weight	Age, sex, brain weight, social status	Age, sex, brain weight

Table S4. Model experimental and control variables. SDI = "Social Dominance Index". "Perc-rank" = Perc R package generated rank *(85)*.

ROI \ Social variables	Betweenness	Closeness	eigenvector centrality
**ACC**	r = -0.003; p = 0.978	r = -0.203; p = 0.126	r = 0.102; p = 0.451
**Amydgala**	r = -0.113; p = 0.398	r = 0.022; p = 0.870	r = 0.041; p = 0.761
**mid-STS**	r = 0.086; p = 0.520	r = -0.189; p = 0.155	r = 0.164; p = 0.223
**PCC**	r = -0.248; p = 0.060	r = -0.236; p = 0.074	r = -0.063; p = 0.643

Table S5. Indirect measures of connectedness do not correlate with the size of four key areas in the social brain network, even after excluding fully disconnected individuals (n=9). Correlation coefficient (r) and uncorrected p-value (p) for relationships between average log-transformed Jacobian value of four social brain network regions of interest (bottom to top: PCC, mid-STS, amygdala, and ACC) and indirect measures of connectedness (columns left to right: betweenness, closeness and eigenvector centrality) in 59 adult macaques. None of the correlations were significant.

REFERENCES AND NOTES

1. M. L. Platt, R. M. Seyfarth, D. L. Cheney, Adaptations for social cognition in the primate brain. *Philos. Trans. R. Soc. Lond. Ser. B Biol. Sci.* **371**, 20150096 (2016).

2. D. Cheney, R. Seyfarth, B. Smuts, Social relationships and social cognition in nonhuman primates. *Science* **234**, 1361–1366 (1986).

3. R. I. M. Dunbar, S. Shultz, Evolution in the social brain. *Science* **317**, 1344–1347 (2007).

4. H. Friedman, N. Ator, N. Haigwood, W. Newsome, J. S. Allan, T. G. Golos, J. H. Kordower, R. E. Shade, M. E. Goldberg, M. R. Bailey, P. Bianchi, The critical role of nonhuman primates in medical research. *Pathog. Immun.* **2**, 352–365 (2017).

5. M. Petrides, F. Tomaiuolo, E. H. Yeterian, D. N. Pandya, The prefrontal cortex: Comparative architectonic organization in the human and the macaque monkey brains. *Cortex* **48**, 46–57 (2012).

6. J. Sallet, R. B. Mars, M. P. Noonan, J. L. Andersson, J. X. O'Reilly, S. Jbabdi, P. L. Croxson, M. Jenkinson, K. L. Miller, M. F. S. Rushworth, Social network size affects neural circuits in macaques. *Science* **334**, 697–700 (2011).

7. R. Kanai, B. Bahrami, R. Roylance, G. Rees, Online social network size is reflected in human brain structure. *Proc. Biol. Sci.* **279**, 1327–1334 (2012).

8. M. P. Noonan, J. Sallet, R. B. Mars, F. X. Neubert, J. X. O'Reilly, J. L. Andersson, A. S. Mitchell, A. H. Bell, K. L. Miller, M. F. S. Rushworth, A neural circuit covarying with social hierarchy in macaques. *PLOS Biol.* **12**, e1001940 (2014).

9. R. M. Sapolsky, The influence of social hierarchy on primate health. *Science* **308**, 648–652 (2005).

10. C. Parkinson, A. M. Kleinbaum, T. Wheatley, Spontaneous neural encoding of social network position. *Nat. Hum. Behav.* **1**, 1–7 (2017).

11. R. B. Mars, F.-X. Neubert, M. P. Noonan, J. Sallet, I. Toni, M. F. S. Rushworth, On the relationship between the "default mode network" and the "social brain". *Front. Hum. Neurosci.* **6**, 189 (2012).

12. M. Schurz, J. Radua, M. G. Tholen, L. Maliske, D. S. Margulies, R. B. Mars, J. Sallet, P. Kanske, Toward a hierarchical model of social cognition: A neuroimaging meta-analysis and integrative review of empathy and theory of mind. *Psychol. Bull.* **147**, 293–327 (2021).

13. L. J. N. Brent, Friends of friends: Are indirect connections in social networks important to animal behaviour? *Anim. Behav.* **103**, 211–222 (2015).

14. J. P. Lerch, A. P. Yiu, A. Martinez-Canabal, T. Pekar, V. D. Bohbot, P. W. Frankland, R. M. Henkelman, S. A. Josselyn, J. G. Sled, Maze training in mice induces MRI-detectable brain shape changes specific to the type of learning. *NeuroImage* **54**, 2086–2095 (2011).

15. A. L. Manera, M. Dadar, D. L. Collins, S. Ducharme; Frontotemporal lobar degeneration neuroimaging initiative, Deformation based morphometry study of longitudinal MRI changes in behavioral variant frontotemporal dementia. *Neuroimage Clin.* **24**, 102079 (2019).

16. J. Holt-Lunstad, T. B. Smith, J. B. Layton, Social relationships and mortality risk: A meta-analytic review. *PLOS Med.* **7**, e1000316 (2010).

17. J. Ostner, O. Schülke, in *Advances in the Study of Behavior*, M. Naguib, L. Barrett, S. D. Healy, J. Podos, L. W. Simmons, M. Zuk, Eds. (Academic Press, 2018), vol. 50, pp. 127–175.

18. B. Seltzer, D. N. Pandya, Afferent cortical connections and architectonics of the superior temporal sulcus and surrounding cortex in the rhesus monkey. *Brain Res.* **149**, 1–24 (1978).

19. J. Sliwa, W. A. Freiwald, A dedicated network for social interaction processing in the primate brain. *Science* **356**, 745–749 (2017).

20. T. Ninomiya, A. Noritake, M. Isoda, Live agent preference and social action monitoring in the macaque mid-superior temporal sulcus region. *Proc. Natl. Acad. Sci. U.S.A.* **118**, e2109653118 (2021).

21. W. S. Ong, S. Madlon-Kay, M. L. Platt, Neuronal correlates of strategic cooperation in monkeys. *Nat. Neurosci.* **24**, 116–128 (2021).

22. L. Roumazeilles, M. Schurz, M. Lojkiewiez, L. Verhagen, U. Schüffelgen, K. Marche, A. Mahmoodi, A. Emberton, K. Simpson, O. Joly, M. Khamassi, M. F. S. Rushworth, R. B. Mars, J. Sallet, Social prediction modulates activity of macaque superior temporal cortex. *Sci. Adv.* **7**, eabh2392 (2021).

23. R. B. Mars, J. Sallet, F.-X. Neubert, M. F. S. Rushworth, Connectivity profiles reveal the relationship between brain areas for social cognition in human and monkey temporoparietal cortex. *Proc. Natl. Acad. Sci. U.S.A.* **110**, 10806–10811 (2013).

24. H. C. Evrard, The organization of the primate insular cortex. *Front. Neuroanat.* **13**, 43 (2019).

25. K. S. Saleem, N. K. Logothetis, *A Combined MRI and Histology Atlas of the Rhesus Monkey Brain in Stereotaxic Coordinates* (Academic Press, 2012).

26. H. C. Evrard, N. K. Logothetis, A. D. B. Craig, Modular architectonic organization of the insula in the macaque monkey. *J. Comp. Neurol.* **522**, 64–97 (2014).

27. F. Caruana, A. Jezzini, B. Sbriscia-Fioretti, G. Rizzolatti, V. Gallese, Emotional and social behaviors elicited by electrical stimulation of the insula in the macaque monkey. *Curr. Biol.* **21**, 195–199 (2011).

28. O. Boucher, I. Rouleau, M. Lassonde, F. Lepore, A. Bouthillier, D. K. Nguyen, Social information processing following resection of the insular cortex. *Neuropsychologia* **71**, 1–10 (2015).

29. C. Lamm, T. Singer, The role of anterior insular cortex in social emotions. *Brain Struct. Funct.* **214**, 579–591 (2010).

30. A. Jezzini, S. Rozzi, E. Borra, V. Gallese, F. Caruana, M. Gerbella, A shared neural network for emotional expression and perception: An anatomical study in the macaque monkey. *Front. Behav. Neurosci.* **9**, 243 (2015).

31. J. Munuera, M. Rigotti, C. D. Salzman, Shared neural coding for social hierarchy and reward value in primate amygdala. *Nat. Neurosci.* **21**, 415–423 (2018).

32. J. T. Klein, M. L. Platt, Social information signaling by neurons in primate striatum. *Curr. Biol.* **23**, 691–696 (2013).

33. J. Bergelson, M. Kreitman, D. A. Petrov, A. Sanchez, M. Tikhonov, Functional biology in its natural context: A search for emergent simplicity. *eLife* **10**, e67646 (2021).

34. C. M. Berman, Mother-infant relationships among free-ranging rhesus monkeys on Cayo Santiago: A comparison with captive pairs. *Anim. Behav.* **28**, 860–873 (1980).

35. C. M. Berman, The ontogeny of social relationships with group companions among free-ranging infant rhesus monkeys I. Social networks and differentiation. *Anim. Behav.* **30**, 149–162 (1982).

36. D. Maestripieri, Maternal influences on primate social development. *Behav. Ecol. Sociobiol.* **72**, 130 (2018).

37. F. B. de Waal, Macaque social culture: Development and perpetuation of affiliative networks. *J. Comp. Psychol.* **110**, 147–154 (1996).

38. S. Kwak, W.-T. Joo, Y. Youm, J. Chey, Social brain volume is associated with in-degree social network size among older adults. *Proc. Biol. Sci.* **285**, 20172708 (2018).

39. S. W. C. Chang, Coordinate transformation approach to social interactions. *Front. Neurosci.* **7**, 147 (2013).

40. D. Maestripieri, C. L. Hoffman, in *Bones, Genetics, and Behavior of Rhesus Macaques: Macaca mulatta of Cayo Santiago and Beyond*, Q. Wang, Ed. (Springer New York, 2012), pp. 247–262.

41. J. H. Manson, Do female rhesus macaques choose novel males? *Am. J. Primatol.* **37**, 285–296 (1995).

42. J. A. Firth, B. C. Sheldon, L. J. N. Brent, Indirectly connected: Simple social differences can explain the causes and apparent consequences of complex social network positions. *Proc. Biol. Sci.* **284**, 20171939 (2017).

43. C. Testard, S. M. Larson, M. Watowich, C. H. Kaplinsky, A. Bernau, M. Faulder, A. Ruiz-Lambides, J. P. Higham, M. Montague, N. Snyder-Mackler, M. L. Platt, L. J. N. Brent, Rhesus macaques build new social connections after a natural disaster. *Curr. Biol.* **31**, 2299–2309.e7 (2020).

44. M. A. Pavez-Fox, J. E. Negron-Del Valle, I. J. Thompson, C. S. Walker, S. E. Bauman, O. Gonzalez, N. Compo, A. Ruiz-Lambides, M. I. Martinez; Cayo Biobank Research Unit, M. L. Platt, M. J. Montague, J. P. Higham, N. Snyder-Mackler, L. J. N. Brent, Sociality predicts individual variation in the immunity of free-ranging rhesus macaques. *Physiol. Behav.* **241**, 113560 (2021).

45. Y. Wang, A. Metoki, K. H. Alm, I. R. Olson, White matter pathways and social cognition. *Neurosci. Biobehav. Rev.* **90**, 350–370 (2018).

46. M. P. Noonan, R. B. Mars, J. Sallet, R. I. M. Dunbar, L. K. Fellows, The structural and functional brain networks that support human social networks. *Behav. Brain Res.* **355**, 12–23 (2018).

47. M. S. Livingstone, J. L. Vincent, M. J. Arcaro, K. Srihasam, P. F. Schade, T. Savage, Development of the macaque face-patch system. *Nat. Commun.* **8**, 14897 (2017).

48. A. Wang, C. Payne, S. Moss, W. R. Jones, J. Bachevalier, Early developmental changes in visual social engagement in infant rhesus monkeys. *Dev. Cogn. Neurosci.* **43**, 100778 (2020).

49. R. A. Hinde, Y. Spencer-Booth, The behaviour of socially living rhesus monkeys in their first two and a half years. *Anim. Behav.* **15**, 169–196 (1967).

50. J. B. Silk, Social components of fitness in primate groups. *Science* **317**, 1347–1351 (2007).

51. S. Ellis, N. Snyder-Mackler, A. Ruiz-Lambides, M. L. Platt, L. J. N. Brent, Deconstructing sociality: The types of social connections that predict longevity in a group-living primate. *Proc. Biol. Sci.* **286**, 20191991 (2019).

52. A. R. DeCasien, J. P. Higham, Primate mosaic brain evolution reflects selection on sensory and cognitive specialization. *Nat. Ecol. Evol.* **3**, 1483–1493 (2019).

53. L. E. Powell, K. Isler, R. A. Barton, Re-evaluating the link between brain size and behavioural ecology in primates. *Proc. Biol. Sci.* **284**, 20171765 (2017).

54. M. J. Kessler, R. G. Rawlins, A 75-year pictorial history of the Cayo Santiago rhesus monkey colony. *Am. J. Primatol.* **78**, 6–43 (2016).

55. R. Hernandez-Pacheco, D. L. Delgado, R. G. Rawlins, M. J. Kessler, A. V. Ruiz-Lambides, E. Maldonado, A. M. Sabat, Managing the Cayo Santiago rhesus macaque population: The role of density. *Am. J. Primatol.* **78**, 167–181 (2016).

56. J. Altmann, Observational study of behavior: Sampling methods. *Behaviour* **49**, 227–266 (1974).

57. L. J. N. Brent, A. Maclarnon, M. L. Platt, S. Semple, Seasonal changes in the structure of rhesus macaque social networks. *Behav. Ecol. Sociobiol.* **67**, 349–359 (2013).

58. L. Kulik, F. Amici, D. Langos, A. Widdig, Sex differences in the development of social relationships in rhesus Macaques (Macaca mulatta). *Int. J. Primatol.* **36**, 353–376 (2015).

59. B. Chapais, M. Girard, G. Primi, Non-kin alliances, and the stability of matrilineal dominance relations in Japanese macaques. *Anim. Behav.* **41**, 481–491 (1991).

60. M. A. Van Noordwijk, C. P. Van Schaik, Male migration and rank acquisition in wild long-tailed macaques (Macaca fascicularis). *Anim. Behav.* **33**, 849–861 (1985).

61. J. P. Higham, D. Maestripieri, The costs of reproductive success in male rhesus Macaques (Macaca mulatta) on Cayo Santiago. *Int. J. Primatol.* **35**, 661–676 (2014).

62. B. Chapais, Dominance, relatedness and the structure of female relationships in rhesus monkeys, in *Primate Social Relationships: An Integrated Approach* (Blackwell, 1983).

63. L. J. N. Brent, S. Semple, C. Dubuc, M. Heistermann, A. Maclarnon, Social capital and physiological stress levels in free-ranging adult female rhesus macaques. *Physiol. Behav.* **102**, 76–83 (2011).

64. G. Csardi, T. Nepusz, The igraph software package for complex network research. *Int. J. Complex Syst.* **1695**, 1–9 (2006).

65. S. Foxley, S. Jbabdi, S. Clare, W. Lam, O. Ansorge, G. Douaud, K. Miller, Improving diffusion-weighted imaging of post-mortem human brains: SSFP at 7T. *NeuroImage* **102**, 579–589 (2014).

66. S. M. Smith, Fast robust automated brain extraction. *Hum. Brain Mapp.* **17**, 143–155 (2002).

67. M. Jenkinson, C. F. Beckmann, T. E. J. Behrens, M. W. Woolrich, S. M. Smith, FSL. *NeuroImage* **62**, 782–790 (2012).

68. A. M. Winkler, G. R. Ridgway, M. A. Webster, S. M. Smith, T. E. Nichols, Permutation inference for the general linear model. *NeuroImage* **92**, 381–397 (2014).

69. C. Gaser, I. Nenadic, B. R. Buchsbaum, E. A. Hazlett, M. S. Buchsbaum, Deformation-based morphometry and its relation to conventional volumetry of brain lateral ventricles in MRI. *NeuroImage* **13**, 1140–1145 (2001).

70. A. D. Leow, I. Yanovsky, M.-C. Chiang, A. D. Lee, A. D. Klunder, A. Lu, J. T. Becker, S. W. Davis, A. W. Toga, P. M. Thompson, Statistical properties of Jacobian maps and the realization of unbiased large-deformation nonlinear image registration. *IEEE Trans. Med. Imaging* **26**, 822–832 (2007).

71. C. Testard, S. M. Larson, M. M. Watowich, C. H. Kaplinsky, A. Bernau, M. Faulder, H. H. Marshall, J. Lehmann, A. Ruiz-Lambides, J. P. Higham, M. J. Montague, N. Snyder-Mackler, M. L. Platt, L. J. N. Brent, Rhesus macaques build new social connections after a natural disaster. *Curr. Biol.* **31**, 2299–2309.e7 (2021).

72. L. J. N. Brent, A. Ruiz-Lambides, M. L. Platt, Persistent social isolation reflects identity and social context but not maternal effects or early environment. *Sci. Rep.* **7**, 17791 (2017).

73. S. M. Smith, T. E. Nichols, Threshold-free cluster enhancement: Addressing problems of smoothing, threshold dependence and localisation in cluster inference. *NeuroImage* **44**, 83–98 (2009).

74. P. M. Kappeler, M. E. Pereira, C. P. van Schaik, in *Primate Life Histories and Socioecology*, P. M. Kappeler, Ed. (University of Chicago Press, 2003), vol. 395, pp. 1–20.

75. C. M. Berman, E. Kapsalis, Development of kin bias among rhesus monkeys: Maternal transmission or individual learning? *Anim. Behav.* **58**, 883–894 (1999).

76. D. Maestripieri, in *Encyclopedia of Evolutionary Psychological Science*, V. Weekes-Shackelford, T. K. Shackelford, V. A. Weekes-Shackelford, Eds. (Springer International Publishing, 2016), pp. 1–4.

77. D. Maestripieri, S. G. Lindell, J. D. Higley, Intergenerational transmission of maternal behavior in rhesus macaques and its underlying mechanisms. *Dev. Psychobiol.* **49**, 165–171 (2007).

78. C. M. Berman, Early agonistic experience and rank acquisition among free-ranging infant rhesus monkeys. *Int. J. Primatol.* **1**, 153–170 (1980).

79. M. E. Pereira, Development and social dominance among group-living primates. *Am. J. Primatol.* **37**, 143–175 (1995).

80. J. H. Sul, L. S. Martin, E. Eskin, Population structure in genetic studies: Confounding factors and mixed models. *PLOS Genet.* **14**, e1007309 (2018).

81. H. M. Kang, N. A. Zaitlen, C. M. Wade, A. Kirby, D. Heckerman, M. J. Daly, E. Eskin, Efficient control of population structure in model organism association mapping. *Genetics* **178**, 1709–1723 (2008).

82. V. Wimmer, T. Albrecht, H.-J. Auinger, C.-C. Schön, synbreed: A framework for the analysis of genomic prediction data using R. *Bioinformatics* **28**, 2086–2087 (2012).

83. Y. Benjamini, Y. Hochberg, Controlling the false discovery rate: A practical and powerful approach to multiple testing. *J. R. Stat. Soc. Ser. B Stat. Methodol.* **57**, 289–300 (1995).

84. R. S. J. Frackowiak, K. J. Friston, C. D. Frith, R. J. Dolan, C. J. Price, S. Zeki, J. T. Ashburner, W. D. Penny, in *Human Brain Function* (Academic Press, ed. 2, 2004), pp. 867–879.

85. B. McCowan, J. Vandeleest, K. Balasubramaniam, F. Hsieh, A. Nathman, B. Beisner, Measuring dominance certainty and assessing its impact on individual and societal health in a nonhuman primate model: A network approach. *Philos. Trans. R. Soc. Lond. Ser. B Biol. Sci.* **377**, 20200438 (2022).

86. S. M. Schwartz, J. W. Kemnitz, Age- and gender-related changes in body size, adiposity, and endocrine and metabolic parameters in free-ranging rhesus macaques. *Am. J. Phys. Anthropol.* **89**, 109–121 (1992).
